# Sialotranscriptomics of *Rhipicephalus zambeziensis* reveals intricate expression profiles of secretory proteins and suggests tight temporal transcriptional regulation during blood-feeding

**DOI:** 10.1186/s13071-017-2312-4

**Published:** 2017-08-10

**Authors:** Minique Hilda de Castro, Daniel de Klerk, Ronel Pienaar, D Jasper G Rees, Ben J Mans

**Affiliations:** 1 Epidemiology, Parasites and Vectors, Onderstepoort Veterinary Research, Agricultural Research Council, Onderstepoort, South Africa; 2 Biotechnology Platform, Agricultural Research Council, Onderstepoort, South Africa; 3 College of Agriculture and Environmental Sciences, University of South Africa, Johannesburg, South Africa; 4 Department of Veterinary Tropical Diseases, Faculty of Veterinary Science, University of Pretoria, Pretoria, South Africa

**Keywords:** *Rhipicephalus zambeziensis*, *De novo* transcriptome assembly, Tick salivary glands, Sialotranscriptomics, Secretory proteins, Next generation sequencing, Differential expression

## Abstract

**Background:**

Ticks secrete a diverse mixture of secretory proteins into the host to evade its immune response and facilitate blood-feeding, making secretory proteins attractive targets for the production of recombinant anti-tick vaccines. The largely neglected tick species, *Rhipicephalus zambeziensis*, is an efficient vector of *Theileria parva* in southern Africa but its available sequence information is limited. Next generation sequencing has advanced sequence availability for ticks in recent years and has assisted the characterisation of secretory proteins. This study focused on the *de novo* assembly and annotation of the salivary gland transcriptome of *R. zambeziensis* and the temporal expression of secretory protein transcripts in female and male ticks, before the onset of feeding and during early and late feeding.

**Results:**

The sialotranscriptome of *R. zambeziensis* yielded 23,631 transcripts from which 13,584 non-redundant proteins were predicted. Eighty-six percent of these contained a predicted start and stop codon and were estimated to be putatively full-length proteins. A fifth (2569) of the predicted proteins were annotated as putative secretory proteins and explained 52% of the expression in the transcriptome. Expression analyses revealed that 2832 transcripts were differentially expressed among feeding time points and 1209 between the tick sexes. The expression analyses further indicated that 57% of the annotated secretory protein transcripts were differentially expressed. Dynamic expression profiles of secretory protein transcripts were observed during feeding of female ticks. Whereby a number of transcripts were upregulated during early feeding, presumably for feeding site establishment and then during late feeding, 52% of these were downregulated, indicating that transcripts were required at specific feeding stages. This suggested that secretory proteins are under stringent transcriptional regulation that fine-tunes their expression in salivary glands during feeding. No open reading frames were predicted for 7947 transcripts. This class represented 17% of the differentially expressed transcripts, suggesting a potential transcriptional regulatory function of long non-coding RNA in tick blood-feeding.

**Conclusions:**

The assembled sialotranscriptome greatly expands the sequence availability of *R. zambeziensis*, assists in our understanding of the transcription of secretory proteins during blood-feeding and will be a valuable resource for future vaccine candidate selection.

**Electronic supplementary material:**

The online version of this article (doi:10.1186/s13071-017-2312-4) contains supplementary material, which is available to authorized users.

## Background

Ticks are hematophagous ectoparasites of a wide range of wild and domestic animals and humans worldwide, and serve as coincidental vectors of a variety of diseases [1, 2]. After mosquitos, ticks are the second most important arthropod vector of human diseases and transmit more bacterial, viral and protozoan pathogens than any other arthropod [3]. Chemical acaricides have always been the most effective mechanism of tick control [4], but the emergence of acaricide-resistance [5] and health concerns due to chemically residues in meat, milk and the environment [6] have shifted the focus of tick biologists to the development of recombinant anti-tick vaccines [7–10]. The prospect of immunising animals by exposure to tick antigens was already acknowledged in the 1970’s when extracted antigens from the tick gut were injected into guinea pigs and cattle, resulting in protection from subsequent tick challenges [11]. Since then a large number of recombinant tick proteins have been investigated and tested for protection efficiencies in hosts, with some showing very promising efficacy values (reviewed in [10]). Even though recombinant anti-tick vaccines have been shown to be attractive for tick control, their practical development has proven difficult as only two anti-tick vaccines have been commercialised to date, both targeting the same *R. microplus* Bm86 gut protein [12, 13]. The search for effective vaccine candidates therefore continues.

Ticks have evolved a complicated cocktail of secretory proteins and molecules that are secreted into the host to evade host immune surveillance and suppress host defence responses, enabling ticks to feed unnoticed until repletion (reviewed in [14–20]). In recent years, it has been realised that tick salivary glands, the organs in closest proximity to the host during feeding and actively producing secretory proteins, are much more diverse than initially anticipated (reviewed in [15, 21]). Previous studies have shown that secretory proteins are under positive selection [22, 23] and have been subjected to gene duplications, resulting in lineage-specific expansions [14–16, 24] and large multi-genic functionally redundant protein families [19, 21]. Large secretory protein families containing functionally redundant members confine vaccine development, where immunisation against a protein in a multi-genic family may be circumvented by the expression of another functionally redundant member in the same family [7]. It is therefore unlikely that secretory proteins will serve as successful targets for vaccine development in the future without a thorough understanding of the secretory protein families involved in blood-feeding and their temporal expression and regulation during feeding phases. Complete sequence datasets of ticks are required to address these caveats. The first completed genome sequence of a tick species, *Ixodes scapularis,* has recently become available from the collaborative effort of a global tick consortium [25, 26]. The large estimated genome sizes of hard tick species, 2.0–7.1 Gbp [27, 28], make whole genome sequencing an unlikely technology to be routinely adopted by tick biologists in the near future. A more realistic strategy for ticks is sequencing of the expressed RNA species in the salivary glands, generating tick sialotranscriptomes, of which a number have become available in recent years [29–39]. The availability of these transcriptomes affords unprecedented insight into tick salivary gland biology and the extensive expansion in secretory protein families. With the transcriptome sequencing of only a handful of the nearly 900 known tick species [1], it can be expected that the knowledge of tick salivary glands will greatly expand in the coming years.

*Rhipicephalus zambeziensis* is distributed through eastern and southern Africa and vectors the protozoan parasite *Theileria parva,* causative agent of East Coast fever, Corridor disease and January disease [40–42]. The main vector of *T. parva*, *R. appendiculatus*, has a much wider distribution throughout central, eastern and southern Africa [42, 43]. *Rhipicephalus zambeziensis* is better adapted to extreme environmental conditions [44] and naturally occurs in hotter and drier regions than *R. appendiculatus* [40]. One of the most pertinent differences between *R. zambeziensis* and *R. appendiculatus* is the variability in vector competence of *T. parva*. During infection experiments, more *R. zambeziensis* ticks were infected and at higher infection loads than *R. appendiculatus* ticks [45–47], indicating, at least experimentally, that *R. zambeziensis* is a better vector of *T. parva*. Furthermore, changes in climatic conditions (e.g. temperature and rainfall) can result in suitable environments in regions not normally part of the natural distribution of a tick species [48]. Due to the projected temperature increase and rainfall decrease in sub-Saharan Africa it has been predicted that environmental regions suitable for *R. appendiculatus* habitation will change [49], resulting in regions better suited for *R. zambeziensis* ticks that prefer hotter and drier conditions [50]. In a similar situation, the competent vector and invasive species *R. microplus* displaces *R. decoloratus* on a wide scale following climatic gradients, resulting in serious tick-borne disease control issues [51]. The risk associated with the expansion of *R. zambeziensis,* a highly competent vector of *T. parva*, to a wider distribution due to projected climatic changes, may therefore have serious implications for the control of East Coast fever, Corridor disease and January disease*.*

Prior to the start of this study, only 33 *R. zambeziensis* sequences (nucleotide and protein) were publically available. To alleviate the sequence shortcomings of *R. zambeziensis*, a representative, high quality sialotranscriptome was assembled *de novo* and annotated from female to male ticks during different feeding stages. The assembled transcriptome was used as reference to determine abundances and differential expression of transcripts and protein families during feeding and between different sexes, with special focus on transcripts predicted to be coding for secretory proteins. To our knowledge this is the first report of a *de novo* transcriptome of *R. zambeziensis* using next generation sequencing. This transcriptome will likely prove invaluable for future comparative studies analysing multi-genic secretory protein phylogenies and to broaden our understanding of tick biology and the secretory proteins involved in blood-feeding and host immune modulation. Further, the transcriptome can be used for proteomic analyses in *R. zambeziensis* and for the future selection of potential vaccine candidates.

## Methods

### Tick feeding and salivary gland dissection

A parasite-free *R. zambeziensis* tick colony is maintained under standard laboratory and tick-rearing protocols at Onderstepoort Veterinary Research Institute [52]. The colony was initiated from ticks collected from vegetation in the Marakele National Park, Limpopo Province, South Africa, in 2010. Adult ticks were fed in feeding bags on disease-free Hereford (*Bos taurus*) cattle and carefully removed at 3 and 5 days after attachment. Unfed ticks, from the same batch of engorged nymphs as the fed ticks, were also processed. Twenty male and twenty female ticks were removed at each time point. Ticks were dissected directly after removal from the bovine and the salivary glands extracted and stabilised in RNAlater (Qiagen, Valencia, CA, USA) according to the manufacturer’s specifications until RNA extraction.

### RNA extraction, library preparation and sequencing

Total RNA was extracted using the RNeasy Protect Mini Kit (Qiagen) and residual genomic DNA removed by *DNase I* digestion (Qiagen). Approximately 2 μg of total RNA was used for library generation in the TruSeq RNA Sample Preparation kit (Illumina, San Diego, CA, USA). Fragment fractions of ±300 bp were excised after agarose gel electrophoreses to prepare the libraries for HiScanSQ 100 bp paired end sequencing. Additionally, a pooled library from an equimolar mixture of all six samples was generated for MiSeq sequencing. This library was prepared from longer RNA fragments by a shortened RNA fragmentation step (changed from 8 to 3 min) in the TruSeq Sample Preparation kit, according to the manufacturer’s specifications. A high molecular weight library fraction (± 600–1000 bp) was excised for subsequent MiSeq sequencing, generating 300 bp paired end reads. Sequencing was performed on the HiScanSQ and MiSeq Illumina instruments at the Biotechnology Platform Sequencing Facility (Agricultural Research Council, South Africa).

### Read quality filtering, *de novo* transcriptome assembly and evaluation

Trimmomatic version 0.32 [53] was used to remove Illumina adaptor sequences and low quality bases from the sequence reads using the parameters; ILLUMINACLIP:TruSeq3-PE-2.fa: 2:30:10 LEADING:20 TRAILING:20 SLIDINGWINDOW:4:20 AVGQUAL:15 MINLEN:50. The SLIDINGWINDOW parameter was adjusted to 7:15 for MiSeq generated sequences. Following quality filtering, paired end MiSeq sequences that overlapped by at least 20 bases, were merged into a single sequence using the CLC Genomics Workbench version 7.5.1 (Qiagen). All quality-filtered and merged reads were pooled together to assemble a single *de novo* transcriptome of *R. zambeziensis* using the Trinity software package version 2.1.1 [54, 55]. Standard k-mer size of 25 was used and a minimum k-mer coverage of two was selected to reduce the incorporation of potential sequencing errors. An expression level threshold of fragments per kilobase of exon per million fragments mapped (FPKM) value of 1 was used to select against lowly expressed transcripts, that possibly represent assembly artefacts or background expression [56–58]. The quality of the assembled transcriptome was evaluated using the Transrate version 1.0.0 software package [59] that estimates the quality of each transcript based on the mapping coverage of sequence reads and the alignment to a closely related reference sequence. The set of well-curated predicted proteins from the sequenced tick genome, *I. scapularis* (IscaW1.4) [26], was used as reference. The Transrate transcript evaluation was used as an additional transcript selection process to remove low quality transcripts and improve the confidence in the final transcriptome.

### Transcriptome annotation

The transcriptome was BLASTx aligned (E-value < E-05) against the following protein databases: the National Center for Biotechnology Information (NCBI) non-redundant (NR) and Transcriptome Shotgun Assembly (TSA-NR) databases (retrieved April 2016), the UniProt Knowledgebases (UniProtKB/TrEMBL and UniProtKB/Swiss-Prot, retrieved July 2016), the *I. scapularis* predicted peptides (IscaW1.4, retrieved July 2016) [26], an Acari database (AcariDB; containing all available mite and tick sequences, as described in [37]) retrieved July 2016, and the EuKaryotic Orthologous Groups (KOG) dataset, retrieved July 2016 [60]. Additionally, the transcriptome was BLASTn searched against the NCBI non-redundant nucleotide (Nt) database (retrieved April 2016). The Blast2GO software package [61] was used to search the NR BLASTx results for Gene Ontology (GO) terms and visualised by the Web Gene Ontology Annotation Plot (WEGO) [62]. Kyoto Encyclopedia of Genes and Genomes (KEGG) orthology (KO) identifiers from the *I. scapularis* genome were assigned to the transcripts using the KEGG Automatic Annotation Server (KAAS) [63]. The protein-coding potential of the transcripts were determined by three software packages: Predictor of lncRNAs and mRNAs based on k-mer scheme v1.2 (PLEK) [64]; Coding Potential Calculator (CPC) [65]; and Coding-Potential Assessment Tool (CPAT) [66].

### Open reading frame prediction and annotation

Translation frames obtained from transcripts with significant BLASTx searches against AcariDB were used to predict putative open reading frames (ORFs) of 240 bp or longer using the OrfPredictor Server [67]. A second round of prediction for transcripts where ORFs were not predicted was performed using the orffinder.pl script [68]. The predicted ORFs were translated into amino acid sequences, BLASTp searched against the AcariDB database and compared to the BLASTx results of the transcripts. In cases where the BLAST results were not similar the ORFs were manually inspected and, where possible, ORFs were predicted using the Expert Protein Analysis System (ExPASy) Translate tool [69]. Predicted proteins were BLASTp searched against the search databases mentioned above as well as against NCBI’s Conserved Domain Database (CDD) [70] and the Pfam database [71]. SignalP 4.1 [72] and Phobius [73] were used to identify putative signal peptide signatures and TMHMM 2.0 [74], to predict the transmembrane topology of the amino acid sequences. ORFs with no BLASTp or domain-based matches were discarded, after which CD-HIT v4.5.4 [75] was used to obtain a final non-redundant set of predicted proteins. These proteins were annotated in a priority order of: AcariDB, NR, UniProtKB/TrEMBL, *I. scapularis* proteins, Pfam database and CDD database. The AcariDB was used to classify the proteins into four main classes: putative secretory, housekeeping, unknown function and no hit proteins (proteins for which no significant matches were found in the database). The final set of predicted proteins was evaluated for completeness by searching for the presence of a conserved set of 1066 arthropod single-copy orthologous proteins using the Benchmarking Universal Single-Copy Orthologs (BUSCO) v2 software [76]. To evaluate whether the methodology pipeline followed in this study produced a representative set of *Rhipicephalus* salivary gland proteins, the predicted *R. zambeziensis* proteins were compared to the predicted proteins of two closely related tick sialotranscriptomes: *R. pulchellus* (NCBI Bioproject PRJNA170743) [34] and *R. appendiculatus* (NCBI Bioproject PRJNA297811) [37]. As a further validation of the assembled transcriptome and predicted proteins, 17 previously characterised *R. appendiculatus* protein sequences [77–84] were used in a BLASTp database to identify putative representatives in the *R. zambeziensis* transcriptome.

### Differential expression analysis

The *de novo* assembled transcriptome of *R. zambeziensis* was used as reference for differential expression analysis, whereby the quality-filtered sequence reads of each time point was analysed against it separately. Additionally, the filtered reads within each sex were combined to perform differential expression analysis between male and female ticks. The Trinity software package contains custom scripts to facilitate differential expression analysis [54, 55]. Initially, the reads were mapped to the transcriptome using Bowtie 2 v2.2.3 [85], followed by abundance estimation with the RNA-Seq by Expectation-Maximization (RSEM) v1.2.31 software package [86] using the trimmed mean of M values (TMM) normalisation [87]. The transcripts per million (TPM) values were determined and used to estimate the relative abundance of a transcript, also referred to as its expression level [88]. Differential expression analysis was performed using the Bioconductor/Empirical analysis of digital gene expression data in R (edgeR) v3.14.0 software package [89], using the fixed dispersion value of 0.4 (as recommended for biologically non-replicated samples), fold change of > 4 and false discovery rate (FDR) *P*-value of < 0.01. Chi-square tests with Bonferroni corrections were used for significance testing in comparisons of protein family expression.

## Results

### *De novo* assembly and validation of the *R. zambeziensis* transcriptome

To assemble the salivary gland transcriptome of *R. zambeziensis*, between 22 and 37 million paired HiScanSQ sequencing reads were generated for each time point, together with about 22 million paired MiSeq reads from a pool of all time points (Additional file 1: Table S1). After adapter trimming and quality filtering, 81% of the HiScanSQ reads were retained in a paired end format and 7% as single ends. Of the MiSeq reads, 31% were retained as paired and 35% as single ends reads. All quality-filtered reads were combined to create a dataset containing approximately 192 million read 1 and 146 million read 2 sequencing reads, which was used for *de novo* assembly of the *R. zambeziensis* transcriptome. In total, 140,703 transcripts were assembled that were filtered based on expression level (FPKM values > 1) and read mapping confidences (as estimated by the transcriptome evaluation software, Transrate, [59]). This resulted in a final transcriptome of 23,631 high confidence transcripts (Table 1). Of the sequenced reads, 94% mapped to the initial and 91% to the final transcriptome. This indicated that the nearly 120,000 transcripts excluded from the final assembly represented less than 3% of the total reads, likely representing mostly low quality transcripts. Furthermore, 79% of the transcripts in the final assembly were near full-length, showing a larger than 75% alignment coverage to their best BLAST matches in the *I. scapularis* protein set. The proteins predicted from the final transcriptome were analysed for assembly and annotation completeness by the BUSCO software package [76]. Eighty-six percent of the conserved proteins were both present and full-length and only 1.7% of the proteins were fragmented and not assembled in full-length copies. These metrics indicated that a high quality *R. zambeziensis* transcriptome was assembled, which was close to completion, mostly full-length and highly representative of the sequence reads from which it was build.

**Table 1:** Summary of the *R. zambeziensis* transcriptome assembly and annotation statistics

Transcriptome statistics	Value^a^
Transcriptome assembly statistics	
Total number of transcripts	23,631
Number of transcripts > 500 bp	19,903
Number of transcripts > 1 kb	13,330
Number of transcripts > 10 kb	80
Shortest transcript length (bp)	201
Longest transcript length (bp)	17,108
Mean length of transcripts (bp)	1793.5
Median length of transcripts (bp)	1193
Transcript N50 (bp)	2807
Total bases in assembly (Mb)	42.4
Ambiguous base calls (Ns)	0
GC content (%)	49
Number of non-redundant predicted proteins	13,584
Transcriptome annotation statistics^b^	
BLASTx against NR	12,756 (54.0%)
BLASTx against UniProtKB/TrEMBL	16,451 (69.6%)
BLASTx against UniProtKB/Swiss-Prot	10,572 (44.7%)
BLASTx against TSA-NR	16,711 (70.7%)
BLASTx against *Ixodes scapularis* predicted peptides	11,804 (50.0%)
BLASTx against EuKaryotic Orthologous Groups (KOG)	9620 (40.7%)
BLASTx against AcariDB	18,245 (77.2%)
Assigned with Gene Ontology (GO) terms^c^	11,360 (48.1%)
Assigned with Enzyme Commission (EC) numbers^c^	3493 (14.8%)
Assigned with KEGG orthology (KO) identifiers^d^	4869 (20.6%)
Annotated in at least one database	18,311 (77.5%)

^a^Value indicating either the number of transcripts, proteins or bases, the transcript length or percentage, as indicated in the table

^b^Number (and %) of transcripts annotated based on significant matches (*E*-value < E-05) against databases as detailed in the Methods section

^c^GO terms and EC numbers assigned with Blast2GO

^d^From the KEGG (Kyoto Encyclopedia of Genes and Genomes) Automatic Annotation Server (KAAS) using the *I. scapularis* genome

### Annotation and characterisation of the *R. zambeziensis* transcriptome and predicted proteins

The assembled *R. zambeziensis* transcriptome was annotated by sequence similarity searches against a number of databases, which resulted in 18,311 transcripts (78% of the transcriptome) obtaining a significant match (*E*-value < E-05) to at least one of the databases (Table 1, complete transcript annotation can be found in Additional file 2: Table S6). Nearly half of the transcripts were assigned Gene Ontology terms, which included 12,805 cellular components, 20,458 biological processes and 14,603 molecular functions (Additional file 3: Figure S1). Based on KOG functional categories, most *R. zambeziensis* transcripts were classified as “Post translational modification, protein turnover, chaperones”, “General function prediction only” or “Signal transduction mechanisms” (Additional file 3: Figure S2). The KEGG pathway analysis revealed that most transcripts belonged to the “Ribosome”, “RNA transport” and “Spliceosome” pathways (Additional file 3: Figure S3). Additionally, alignment against the NCBI Nt database retrieved full-length copies of all 4 ribosomal RNA molecules in the *R. zambeziensis* transcriptome (Additional file 2: Table S6).

In total, 15,737 open reading frames (ORFs) were predicted from 66% (15,684) of the *R. zambeziensis* transcripts. A small fraction of the transcripts (0.2%) encoded for more than one ORF. The predicted ORFs were translated into amino acid (aa) sequences and reduced, based on 100% aa sequence similarity, to a non-redundant set of 13,584 predicted proteins (predicted protein annotation can be found in Additional file 4: Table S7). Eighty-six percent of these were estimated to be putatively full-length proteins as they contained both a predicted start and stop codon. Of the transcripts for which no ORFs could be predicted (7947 transcripts), 98% were assigned as putative non-coding sequences by at least two coding potential databases (Additional file 2: Table S6). Signal peptide signatures were observed in 3488 predicted proteins, 2169 proteins contained transmembrane domains and 8061 proteins showed similarity to at least a single Pfam domain (the 30 most abundant R. *zambeziensis* Pfam domains can be found in Additional file 3: Figure S4). AcariDB was used to classify the predicted proteins into: 8139 housekeeping, 2569 secretory, 1706 unknown function and 1170 no hit proteins. Protein annotation was performed based on a priority database order, as set out in the Methods section. Finally, based on the invertebrate mitochondrial genetic code, full-length gene copies of all 13 *R. zambeziensis* mitochondrial proteins were predicted and annotated (Additional file 4: Table S7).

### Comparison of the *R. zambeziensis* transcriptome to other *Rhipicephalus* sialotranscriptomes

**Fig. 1:**
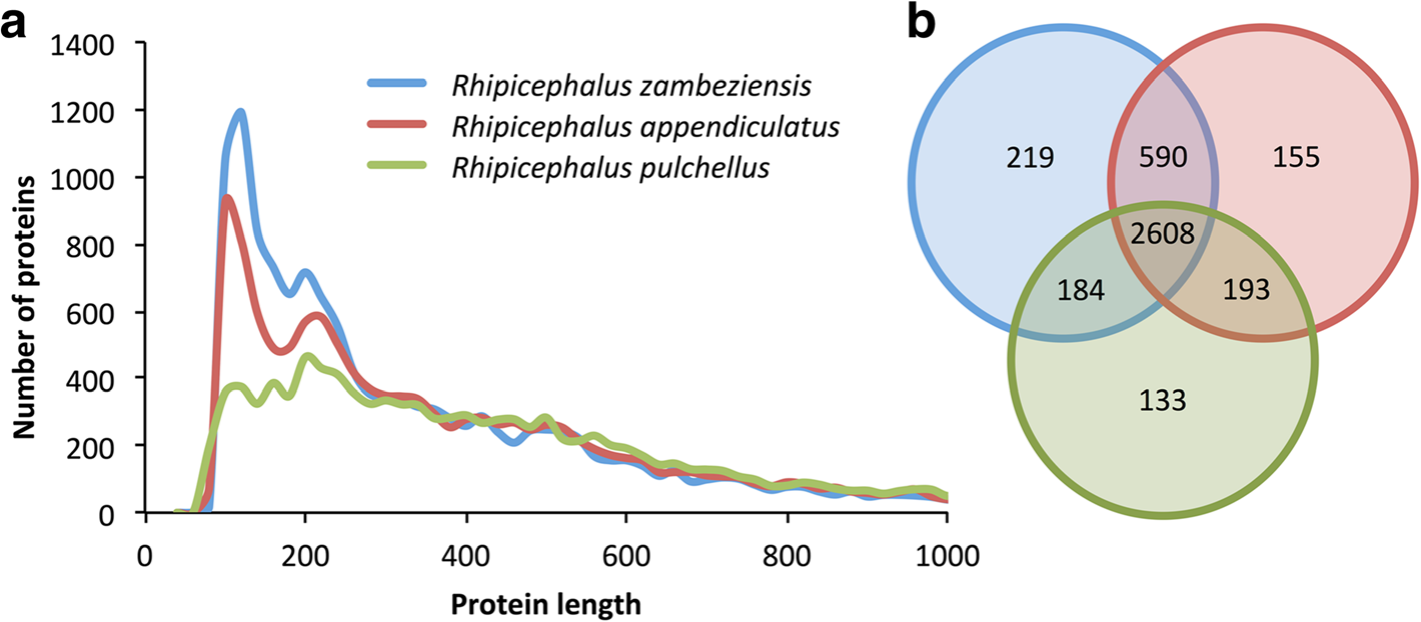
Comparisons of the predicted *R. zambeziensis* proteins to proteins of two closely related *Rhipicephalus* species. **a** Length distribution of the predicted proteins of *R. zambeziensis*, *R. appendiculatus* and *R. pulchellus*. The number of proteins is indicated based on a protein length sliding window of 20 amino acids (aa), showing a maximum length of 1000 aa. **b** Pfam domain comparison of the three *Rhipicephalus* species. Datasets used: 13,584 predicted proteins from the assembled *R. zambeziensis* transcriptome, 12,761 *R. appendiculatus* proteins [37] and 11,227 *R. pulchellus* proteins [34]. *Blue* represents *R. zambeziensis*; *red*, *R. appendiculatus*; and *green*, *R. pulchellus*

The predicted proteins from the assembled *R. zambeziensis* transcriptome were compared to publically available transcriptomes of two closely related *Rhipicephalus* species, for which protein predictions were available. The number of predicted *R. zambeziensis* proteins (13,584) were similar to the number of predicted proteins in the *R. appendiculatus* (12,761) and *R. pulchellus* (11,227) transcriptomes. The lengths of the *R. zambeziensis* proteins (57–4928 aa, average 341 aa) were slightly shorter than those predicted for *R. appendiculatus* (70–4966 aa, average 400 aa) and *R. pulchellus* (66–6645 aa, average 471 aa). However, from the length distribution of the predicted proteins it was evident that the *R. zambeziensis* transcriptome was enriched for shorter proteins without compromising the distribution of longer proteins (Fig. 1a). Pfam domain prediction and comparison among the species showed that most of the domains (64%) were shared among all the transcriptomes (Fig. 1b). More domains were shared between the phylogenetically closer species, *R. zambeziensis* and *R. appendiculatus* (15%), than *R. pulchellus* and any of the two species (about 5% shared with each species). These results showed that the assembled *R. zambeziensis* transcriptome and resulting predicted proteins were comparable to published tick sialotranscriptomes and therefore representative of proteins expected in the salivary glands of feeding *Rhipicephalus* ticks.

### Search for putative functional orthologues in the *R. zambeziensis* transcriptome

To verify that the assembled *R. zambeziensis* transcriptome could be used as a resource in future, we searched for the presence of representatives of 17 previously characterised *R. appendiculatus* proteins. A number of functional studies have been performed in *R. appendiculatus*, and based on its close relation to *R. zambeziensis,* it was chosen as target for the homology searches. Representative *R. zambeziensis* sequences were identified for 13 *R. appendiculatus* proteins based on more than 70% protein identity to the target sequences (Additional file 1: Table S2). Nine putative orthologues were obtained when the minimum protein identity was increased to 90%, representing the identification of a putative orthologous sequence for more than half (53%) of the targeted sequences. These included the: Immunoglobulin G-binding proteins A-C (IGBP-MA-C) [77]; Female-specific histamine-binding protein 1 (HBP1) [78]; *R. appendiculatus* serine proteinase inhibitor serpins 1–3 (RAS-1-3) [81]; *Rhipicephalus* immuno-dominant molecule 36 (RIM36) [79]; and Japanin-like-RA1 precursor (JL-RA1) [84]. Four *R. zambeziensis* proteins shared less than 70% protein identity to their best match, indicating either that these proteins were not assembled in the *R. zambeziensis* transcriptome, that *R. zambeziensis* does not contain orthologues of these proteins or that protein divergence in these families changed the sequences considerably. It is of importance to mention that actual protein function can only be determined by functional protein characterisation, which remains to be performed for the *R. zambeziensis* proteins. Then, except for RIM36 that was assembled in two separate non-overlapping transcripts and RAS-2 that contained no predicted stop codon, all the other *R. zambeziensis* proteins contained both a predicted start and stop codon and were predicted to be full-length. This indicated that the *R. zambeziensis* transcriptome is a valuable resource from which full-length sequences of potential vaccine candidates can be selected in future.

### Expression composition in the feeding phases and sexes of the *R. zambeziensis* transcriptome

**Fig. 2:**
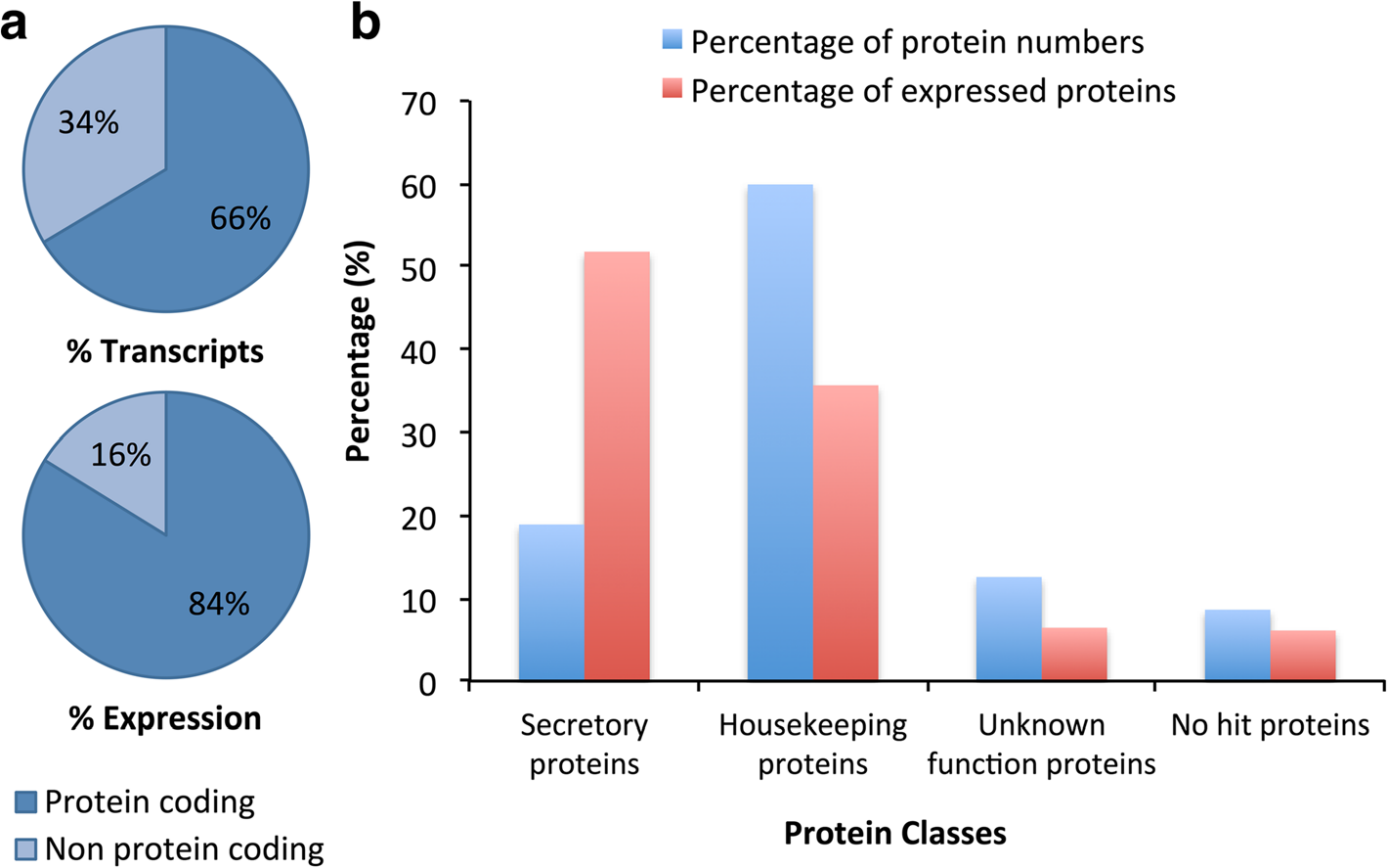
Classification and expression analysis in the *R. zambeziensis* transcriptome. **a** Proportions of predicted protein-coding (indicated by *dark blue* colouring) and predicted non protein-coding (*light blue*) transcripts and their contribution to total expression. **b** Proportions of protein numbers (*blue*) and expression contribution (*red*) of the four predicted protein classes to the total protein-coding fraction of the transcriptome. Expression was estimated by transcripts per million (TPM)

**Fig. 3:**
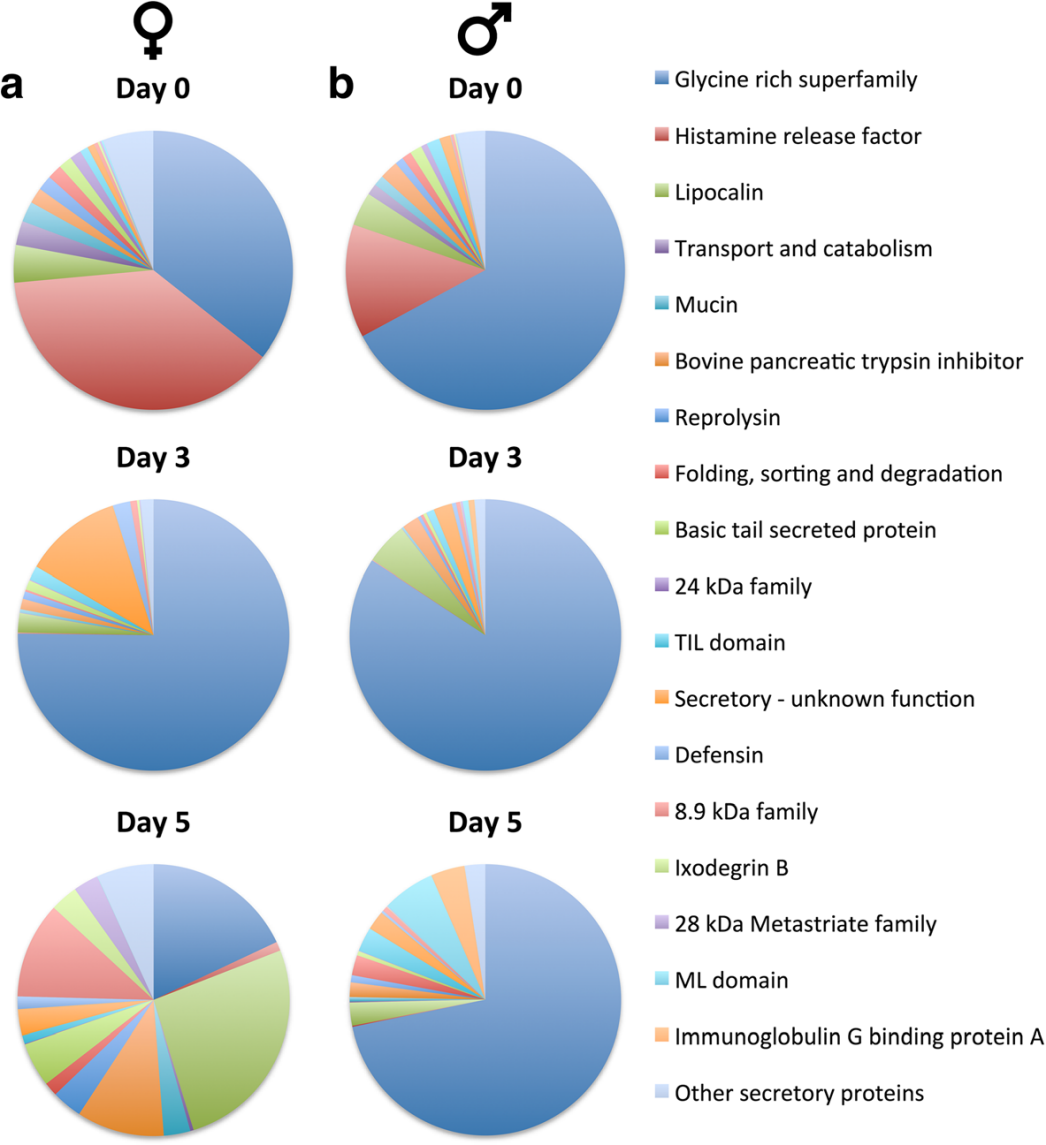
Expression proportions of the *R. zambeziensis* secretory protein families during feeding. The proportions of the highest contributing secretory protein families of female (**a**) and male (**b**) ticks at different feeding time points are indicated. Expression levels were measured by transcripts per million (TPM). Colour key representing the protein families is indicated. Expression values can be found in Additional file 1: Table S3

Between 19 and 30 million clean, paired sequence reads were obtained for each time point after quality filtering (Additional file 1: Table S1) and mapped to the assembled transcriptome of *R. zambeziensis* to estimate transcript abundance. Suitable mapping rates of around 90% were achieved for each time point. Overall, a wide expression range of 0.4–46,919 TPM was observed in the *R. zambeziensis* transcriptome and only a few transcripts (560 transcripts, 2.4% of the transcriptome) accounted for 80% of the total expression (Additional file 2: Table S6). Two thirds of the assembled transcripts were predicted to be protein-coding and represented 84% of the expression in the transcriptome (Fig. 2a). Only a small fraction of the predicted proteins were classified as secretory proteins (19%; 2569 proteins), but this protein class represented more than half of the transcription in the coding fraction of the salivary glands (52%, Fig. 2b). Conversely, the largest protein class at 60%, the housekeeping proteins, represented only 36% of the transcript expression. Within the secretory protein class, 71% of the expression was as a result of a single protein family, the Glycine rich superfamily (Table 2). Some of the other large contributors to the secretory protein class were the Lipocalin (5.6%), Secretory - unknown function (5.0%) and Bovine pancreatic trypsin inhibitor (2.5%) families. Further examination of the secretory protein families showed that their expression composition changed greatly over time, resulting in unique secretory protein compositions for each time point in female and male ticks (Fig. 3a, b, Additional file 1: Table S3). The Glycine rich superfamily was the most abundant family in 4 of the 6 time points, where it ranged from 67 to 84% of the expression, reaching a maximum contribution to expression at the third day of feeding in each sex. In unfed females the most predominant family was the Histamine release factor (HRF) family (38%) and in day 5 fed females, Lipocalin was the most abundant (26%, Fig. 3a). Notably, the HRF family that contributed largely to the expression in the salivary glands of unfed female and male ticks consisted of only a single family member (Rzam_Mc198). At a TPM value of 6057, this transcript was the 20th highest expressed transcript in the *R. zambeziensis* transcriptome.

**Table 2:** Characterisation of the secretory protein families in the *R. zambeziensis* transcriptome

Secretory protein family	Number of family members	Proportion of family members (%)	Average TPM^a^ value	Sum of the family expression (TPM^a^)	Proportion of family expression (%)
Lipocalin	588	22.90	39.88	23,452.37	5.62
Bovine pancreatic trypsin inhibitor	307	11.95	34.07	10,458.51	2.51
Reprolysin	213	8.29	19.36	4123.57	0.99
TIL domain	177	6.89	38.05	6735.14	1.61
Glycine rich superfamily	161	6.27	1842.35	296,618.84	71.07
Basic tail secreted protein	111	4.32	43.04	4777.46	1.14
8.9 kDa family	91	3.54	74.27	6758.57	1.62
Digestive system (including Serine proteases)	87	3.39	9.57	832.96	0.20
Mucin	62	2.41	47.14	2922.86	0.70
28 kDa Metastriate family	56	2.18	31.67	1773.45	0.42
Evasin	55	2.14	33.33	1832.92	0.44
Secretory - unknown function	46	1.79	455.62	20,958.56	5.02
Folding, sorting and degradation (including Cathepsins)	46	1.79	88.22	4058.19	0.97
Cystatin	45	1.75	17.27	777.09	0.19
Gluzincin	43	1.67	7.34	315.45	0.08
Serpin	33	1.29	6.30	207.96	0.05
Ixodegrin B	32	1.25	55.31	1770.02	0.42
One of each family	28	1.09	3.71	103.68	0.02
Carboxypeptidase inhibitor	27	1.05	16.01	432.37	0.10
Chitin-binding proteins	26	1.01	20.30	527.89	0.13
5′-Nucleotidase	25	0.97	6.56	163.87	0.04
Transport and catabolism	24	0.93	35.23	845.47	0.20
24 kDa family	21	0.82	24.99	524.87	0.13
7 dB family	19	0.74	11.34	215.53	0.05
DA-P36 family	19	0.74	10.15	192.90	0.05
Defensin	19	0.74	211.69	4022.20	0.96
ML domain	17	0.66	417.39	7095.58	1.70
Antigen 5 family	14	0.55	57.55	805.64	0.19
Microplusin	14	0.55	47.61	666.59	0.16
8 kDa Amblyomma family	13	0.51	24.52	318.79	0.08
Sphingomyelinase	11	0.43	2.93	32.22	0.01
Glycan biosynthesis and metabolism	10	0.39	9.36	93.63	0.02
Lipid metabolism	10	0.39	5.88	58.76	0.01
Transcription	10	0.39	3.75	37.52	0.01
Translation	8	0.31	10.62	84.92	0.02
Serine/threonine protein kinase	8	0.31	6.59	52.70	0.01
Carbohydrate metabolism	8	0.31	3.53	28.21	0.01
Thyropin	7	0.27	22.67	158.66	0.04
Fibrinogen-related domain	7	0.27	20.84	145.85	0.03
Glutathione metabolism	7	0.27	16.63	116.41	0.03
Metalloprotease	7	0.27	8.28	57.93	0.01
Dermacentor 9 kDa expansion	6	0.23	11.81	70.86	0.02
Replication and repair	6	0.23	5.56	33.37	0.01
Immunoglobulin G binding protein A	5	0.19	989.88	4949.38	1.19
Phospholipase A2	5	0.19	9.54	47.69	0.01
Kazal/vWf domain	4	0.16	4.78	19.11	0.005
Hirudin	3	0.12	108.72	326.17	0.08
SALP15/Ixostatin	3	0.12	35.45	106.35	0.03
14 kDa family	3	0.12	9.75	29.25	0.01
Kazal domain	3	0.12	9.28	27.85	0.01
Signal transduction	3	0.12	2.23	6.68	0.002
Madanin	2	0.08	153.53	307.05	0.07
Energy metabolism	2	0.08	13.13	26.25	0.01
CDIV	2	0.08	9.44	18.88	0.005
EF hand domain	2	0.08	5.33	10.65	0.003
Histamine release factor	1	0.04	6057.33	6057.33	1.45
Fatty acid-binding protein	1	0.04	70.19	70.19	0.02
Kazal/SPARC domain	1	0.04	70.11	70.11	0.02
Immune system	1	0.04	21.85	21.85	0.01
Cysteine rich hydrophobic domain 2	1	0.04	10.83	10.83	0.003
26 kDa family	1	0.04	5.49	5.49	0.001
Proline rich	1	0.04	5.09	5.09	0.001
Cysteine rich	1	0.04	2.23	2.23	0.001

^a^TPM (transcripts per million) values were used to estimate expression

### Differential expression in the *R. zambeziensis* salivary glands

**Fig. 4:**
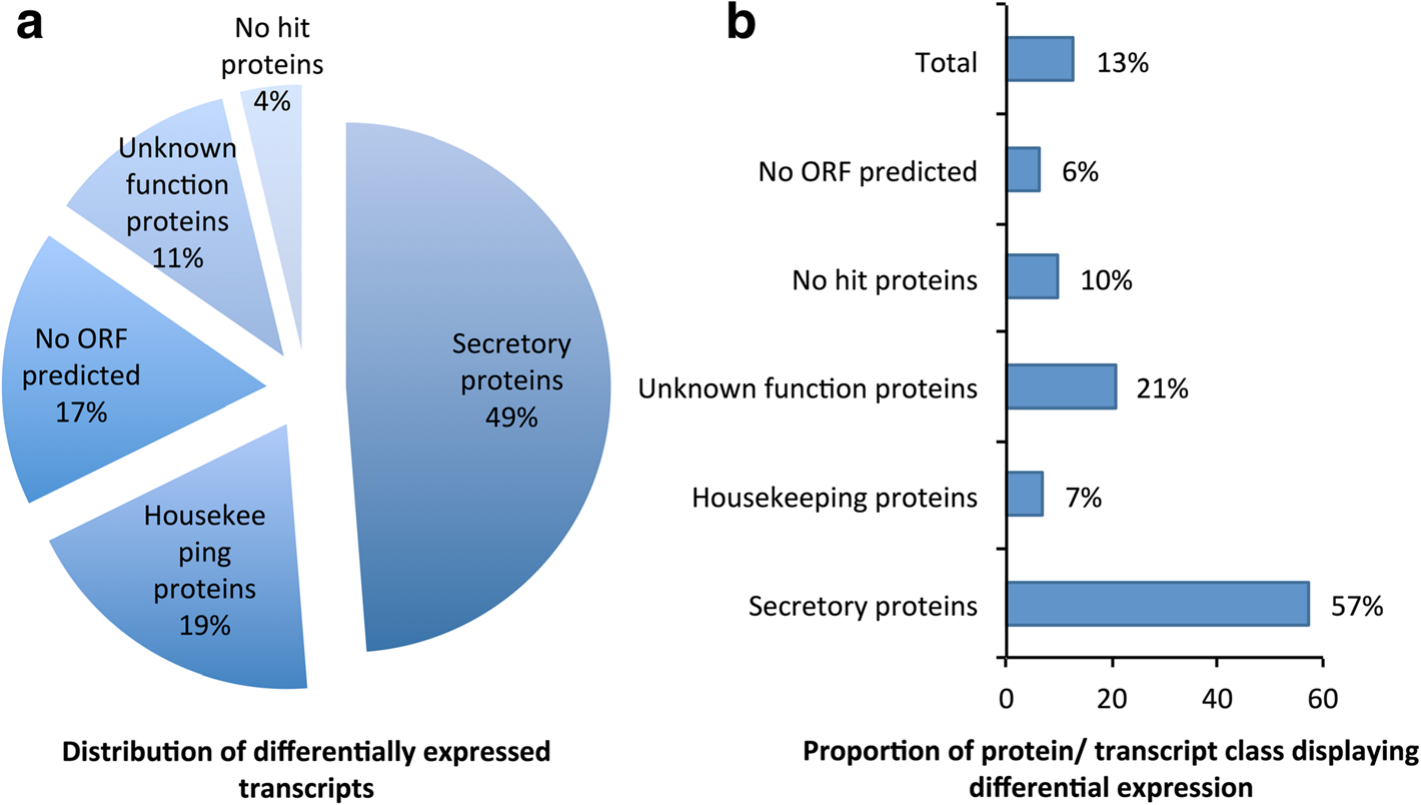
Overview of differential expression in the *R. zambeziensis* sialotranscriptome. **a** Classification of differentially expressed transcripts into different protein or transcript classes. **b** Proportion of differential expression observed within each protein or transcript class. Differential expression analyses were performed using the edgeR software package (with the parameters: fixed dispersion = 0.4, fold change > 4 and FDR *P* < 0.01)

Pairwise comparisons between time points within each sex identified 2832 significantly differentially expressed transcripts, of which 1927 were specific to the feeding phases of female ticks, 663 were male feeding-specific and 242 were shared between the feeding phases of the sexes. A between-gender comparison was performed by combining the reads of all the time points in each sex prior to mapping and expression analysis. This resulted in 1209 differentially expressed transcripts, of which 641 were upregulated in females and 568, in males. Half of the differentially expressed transcripts (1470; 49%) were classified as belonging to the secretory protein class. The remaining 572 (19%), 350 (11%), 113 (4%) and 510 (17%) transcripts belonged to the housekeeping, unknown function, no hit or no ORF classes, respectively (Fig. 4a). The secretory protein families with the largest number of differentially expressed transcripts were the Lipocalin (342 transcripts; 23% of the differentially expressed transcripts in the secretory protein class), Bovine pancreatic trypsin inhibitor (184; 13%), Reprolysin (134; 9%), and Glycine rich (93; 7%) families. Remarkably, 57% of the transcripts classified as secretory proteins were differentially expressed in the salivary glands of *R. zambeziensis* (Fig. 4b), indicating that ticks use a large repertoire of secretory proteins during feeding. Much smaller proportions, 6–21%, of the other classes showed variation in expression and in total 13% of the assembled transcripts were differentially expressed. Furthermore, differentially expressed transcripts accounted for a substantial contribution (45%) of the total expression observed in the salivary glands. This was more pronounced in the secretory protein class, where 72% of the expression was as a result of differentially expressed transcripts. This suggested that the variation observed in the expression composition of the secretory protein families during feeding (Fig. 3) is under strict transcriptional regulation that continually fine-tunes the expression in the salivary glands.

Of the differentially expressed transcripts identified in the between-gender comparison, 376 female and 259 male transcripts were classified as belonging to the secretory protein class (Additional file 1: Table S4). Significantly more transcripts of the Lipocalin (140 female *vs* 42 male transcripts), 8.9 kDa (36 *vs* 12), Reprolysin (27 *vs* 3) and 28 kDa Metastriate (23 *vs* 2) families were upregulated in females after implementing a Bonferroni corrected *P* < 0.0013. In males, the TIL (Trypsin Inhibitor-like) domain (4 *vs* 29), Folding, sorting and degradation (including Cathepsins; 1 *vs* 17), Digestive system (including Serine proteases; 0 *vs* 41), Cystatin (0 *vs* 26) and 7 dB (0 *vs* 13) families showed significantly more upregulated transcripts when compared to females. Of these, the Digestive system, Cystatin and 7 dB families exhibited male-specific upregulation, as no members of these families were upregulated in females. A number of other secretory families showed large differences between the sexes and even gender-specific upregulation, albeit not significant after Bonferroni correction.

### Dynamic expression patterns of the *R. zambeziensis* secretory protein families

**Fig. 5:**
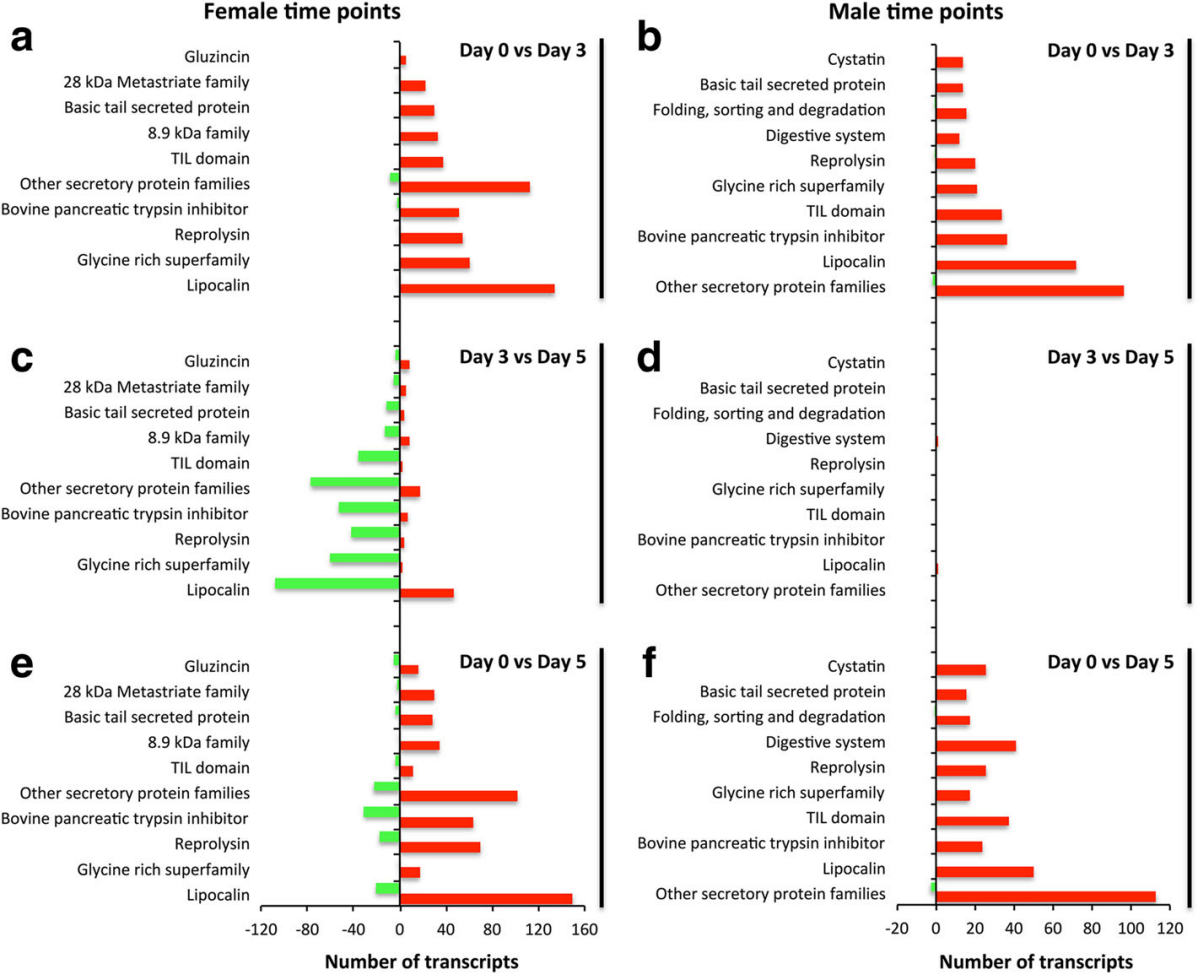
Differentially expressed transcripts of secretory protein families in *R. zambeziensis*. The numbers of up- (red colour) and downregulated (green) secretory protein transcripts, after pairwise comparisons between different feeding time points, are represented. Pairwise comparisons are shown for female: (**a**) day 0 *vs* day 3, (**c**) day 3 *vs* day 5 and (**e**) day 0 *vs* day 5; and male ticks: (**b**) day 0 *vs* day 3, (**d**) day 3 *vs* day 5 and (**f**) day 0 *vs* day 5. The pairwise comparisons are represented as a progression of feeding and show how transcript expression changed from the earlier to the later time point. The edgeR software package (fixed dispersion = 0.4, fold change > 4 and FDR *P* < 0.01) was used for differential expression

Many transcripts belonging to secretory protein families were significantly upregulated during the early tick feeding phase (from unfed to day 3 fed ticks), in both female (541 transcripts) and male (335) ticks (Fig. 5a, b, Additional file 1: Table S5). When considering late feeding (feeding progression from day 3 to 5), in specifically females, most of the differentially expressed secretory transcripts were downregulated (Fig. 5c). Surprisingly, about 52% of these downregulated transcripts were the same transcripts that were upregulated during early feeding, implying that certain secretory transcripts were only required during early feeding stages in females. Hardly any significant differential expression was observed in the male late feeding phase (Fig. 5d), demonstrating that the male day 3 and 5 time points were highly similar in their secretory family expression. Comparisons between unfed and day 5 fed female and male ticks revealed a large number of upregulated secretory transcripts in both sexes, together with some downregulated transcripts in females (Fig. 5e, f). Only about 42% of these upregulated transcripts in female ticks were shared with the transcripts upregulated during early feeding (day 0–3), likely due to more than half of the transcripts upregulated in early feeding undergoing downregulation in female late feeding. Conversely, in males, due to the few significant expression differences observed between day 3 and 5, a larger overlap of 74% was seen between the upregulated transcripts in the day 0–3 and day 0–5 comparisons. Non-significant expression differences between male day 3 and day 5, which would not be picked up by our analyses, might explain the differences observed in the upregulation profiles of the day 0–3 and day 0–5 comparisons in males (Fig. 5b, f). Furthermore, comparisons between the number of upregulated transcripts in each secretory family between day 0–3 and day 0–5 revealed that significantly more transcripts of the Glycine rich (61 *vs* 17 transcripts, *χ* ^2^ = 24.82, *df* = 1, *P* < 0.0001) and TIL domain (38 *vs* 11, *χ* ^2^ = 14.88, *df* = 1, *P* = 0.00011) families were upregulated in earlier feeding points in females (Fig. 5a, e, Additional file 1: Table S5). Also, significantly more of the Digestive system (including Serine proteases, 12 *vs* 41, *χ* ^2^ = 15.87, *df* = 1, *P* < 0.0001) transcripts were upregulated in later feeding points in males (Fig. 5b, f). These results suggested that in tick salivary glands, genes of the secretory protein class are temporally regulated to alter the protein cocktail that is secreted into the host as feeding progresses.

## Discussion

The main aim of this study was the *de novo* assembly, annotation and characterisation of the sialotranscriptome of *R. zambeziensis*, a vector of *T. parva*. Thus far, *R. zambeziensis* has been a largely neglected tick species due to its limited distribution through eastern and southern Africa [40], which was reflected by the small number of publically available sequences of *R. zambeziensis* prior to the start of this study; only 31 nucleotide and 2 protein sequences available in GenBank. In the present study this shortfall was alleviated by the transcriptome assembly and deposition of 13,584 annotated predicted proteins of *R. zambeziensis* into the public domain. This set of predicted proteins is a valuable resource for future vaccine candidate selection in *R. zambeziensis,* as was shown by the availability of mostly full-length versions of previously characterised proteins. The *R. zambeziensis* transcriptome was constructed from sequence reads from male and female ticks, unfed and representative phases of early and late feeding ticks, to represent a large proportion of genes involved in adult tick feeding. Great care was taken to assemble a high quality *R. zambeziensis* transcriptome that was representative of the reads from which it was assembled, near complete, containing a large proportion of full-length protein sequences and similar to closely related tick sialotranscriptomes of *R. pulchellus* [34] and *R. appendiculatus* [37]. Indeed, the number of predicted protein sequences in the *R. zambeziensis* transcriptome was similar to the 11,105 genes predicted to be expressed in the salivary glands of *I. scapularis,* the only tick with a completely sequenced genome [25, 26]. Provisional functions were assigned to the assembled *R. zambeziensis* transcriptome such as histamine [78] and immunoglobulin [77] binding, serine proteinase inhibition [81] and immunomodulation [84]. These functions have been predicted based on high protein identity (≥ 90%) to previously characterised *R. appendiculatus* proteins, but will require functional studies to be confirmed in *R. zambeziensis*. Furthermore, the predicted *R. zambeziensis* proteins were annotated and 19% were classified as putative secretory proteins, in accordance with 13–37% annotated in previously assembled tick sialotranscriptomes [23, 29–31, 33, 34, 37–39]. In total, 8139 of the predicted proteins were classified as putative housekeeping proteins, similar to the number of proteins identified in other metastriate transcriptomes [29, 31, 37] and the predicted number of core housekeeping genes in Chelicerata species, approximately 7000 [20]. Full-length sequences of all 13 mitochondrial genes and all four rRNA molecules were also assembled and can be used in future phylogenetic analyses.

The sequence reads of each time point were independently mapped to the assembled transcriptome to estimate transcript abundance during feeding. During RNA extraction, all ticks in a time point were pooled to obtain enough tissue for extraction and sequencing, similar to methodology followed in other tick transcriptomic studies [23, 30, 32, 33, 35, 36, 90]. The edgeR software package can accommodate non-replicated samples using strict parameters [89], resulting in the identification of fewer false positives but also fewer differentially expressed transcripts. Using these stringent parameters, only transcripts differing by more than a 29-fold change were assigned as differentially expressed in the *R. zambeziensis* transcriptome. Consequently, only large expression differences were considered significant within this work, resulting in high confidence in the assigned differentially expressed transcripts but also the possibility that important feeding genes might have been missed if they had smaller variable expression levels. Nevertheless, 13% of the assembled transcripts showed differential expression and these accounted for nearly half (45%) of the expression in the salivary glands.

In this work, we were mainly interested in the transcriptional response of secretory proteins to tick feeding and we therefore investigated them in greater detail. While the secretory protein class represented only 19% of the predicted proteins in the *R. zambeziensis* salivary glands, this class accounted for the majority of transcript expression (52%). Similar transcript abundance of a small proportion of secretory proteins was seen in other tick sialotranscriptomes: 17 and 63% of the proportions of, respectively, predicted proteins and expression were classified as secretory proteins in *R. appendiculatus* [37]; 23 and 62% in *Hyalomma excavatum* [39]; and 37 and 49% in *Amblyomma americanum* [33]. Nearly half (49%) of the differentially expressed transcripts in *R. zambeziensis* were classified as secretory proteins. Previous studies have also shown that the majority of differentially expressed transcripts in tick salivary glands are classified as secretory proteins [23, 33]. The abundance of transcripts coding for secretory proteins in tick salivary glands was not unexpected as the salivary glands are in primary contact with the host into which secretory proteins are actively being secreted to escape host haemostasis, inflammation and immune response. Remarkably, most (57%) of the predicted *R. zambeziensis* secretory proteins showed significant differential transcript expression, even using the stringent parameters used in this study, and accounted for 72% of the expression observed in the secretory proteins class. These large variations in the *R. zambeziensis* secretory protein expression resulted in each time point resembling a unique transcriptome with different expression proportions of secretory protein families. These differences were more pronounced in female ticks that require more on-host time to feed to repletion. Similarly, changes in secretory protein family compositions during feeding were seen in the saliva of *R. microplus* [91] and *I. scapularis* [92].

Differential expression analysis of the *R. zambeziensis* secretory protein families revealed dynamic profiles of transcript expression during feeding of female ticks. Most of the differentially expressed secretory transcripts were upregulated during early feeding whereas during late feeding most were downregulated. A similar expression pattern was also observed in *A. americanum* salivary glands [93]. However, in *R. zambeziensis* 52% of the upregulated transcripts in early feeding were shared with the downregulated transcripts in late feeding, indicating that these transcripts were specifically required only during early feeding and the establishment of the feeding site in female ticks. Further assessment of the expression regulation in the most differentially expressed secretory protein families (Lipocalin, Bovine pancreatic trypsin inhibitor, Reprolysin and Glycine rich), revealed similar patterns of intricate transcript up- and downregulation and a trend towards preferential expression of transcripts in a single time point in female salivary glands (data not shown). Correspondingly, members of multi-gene secretory protein families showed remarkable differential expression in the salivary glands of *A. americanum* [33, 94] and *I. ricinus* [23], where certain transcripts were uniquely expressed during different developmental stages or feeding time points. Similar profiles were described in *I. scapularis* ticks based on proteomic analyses of saliva collected in 24-h intervals during feeding [92], indicating that transcriptomic profiles of salivary glands are transferable to the proteome being secreted into the host. These results suggest that members of tick secretory protein families are under tight transcriptional regulation that result in complex compositions of secretory proteins in different feeding phases, equipping ticks with the diversity in secretory proteins to evade host immune defences. Indeed, the authors of the above-mentioned studies refer to the expression dynamics observed in tick salivary glands as ‘sialome switching’ or a form of antigenic variation of secretory proteins to evade host immune recognition while still maintaining host immune modulation to feed successfully. Antigenic variation is a mechanism by which infecting microorganisms systematically alter or ‘switch’ their surface proteins to remain unnoticed by the host immune defences (reviewed in [95]). In ticks, antigenic variation will rely on functional redundancy of the multi-genic secretory protein families, where alternate members could be sequentially expressed to evade immune recognition but still retain the same function or a number of members could be expressed concurrently to result in an additive functional effect while maintaining low immunogenicity to each protein [21]. The ability of ticks to preserve crucial blood-feeding functionalities while simultaneously evading the host immune response by altering exposed antigens, make ticks remarkably well adapted to feed for extended periods of time on their hosts.

The above-mentioned conclusions were based on results generated from female ticks [23, 33, 92, 94]. Both female and male ticks were analysed in the current *R. zambeziensis* sialotranscriptome, showing that the expression profiles of secretory proteins were much less complex in males compared to females during feeding. Similar to the low expression variation in male *A. americanum* ticks during feeding [93], only five differentially expressed transcripts were observed between the third and fifth day of feeding of male *R. zambeziensis* ticks, indicating that the male sialotranscriptome necessary for successful feeding had mostly already been established by the third day of feeding. Furthermore, a number of transcripts were differentially expressed between female and male ticks, indicating that some secretory protein families were differentially regulated between the sexes and that female and male ticks feed and evade host immune defences using different mechanisms. This was similar to observations made before [34, 37, 93, 96]. Male ticks feed by continuously attaching and de-attaching from the host to be in close proximity to female ticks [3] in order to mate and assist with establishing a stable feeding cavity from where females can feed to repletion to complete their life-cycles. This might mean that male ticks need a constant supply of secretory proteins to be ready to feed at any time or to secrete decoy antigens into the host to assist females during feeding, which has been shown for male-specific Immunoglobulin-binding proteins [97]. Furthermore, the Serine protease, Cathepsin and Cystatin families, were found to be nearly exclusively upregulated in male *R. zambeziensis* salivary glands when compared to females, similar to families found upregulated in males in previous studies [34, 37]. During mating, male ticks salivate on their spermatophores before inserting them into the female genital pore using their mouthparts [98]. Proteases and protease inhibitors have abundantly been found in insect seminal fluid [99, 100] and the upregulation of their transcripts in male salivary glands might suggest an additional function for these secretory proteins in tick reproduction (as proposed by Tan et al. [34]).

In order to alter the exposed antigens presented to their hosts and to feed unnoticed until repletion, ticks must have evolved stringent temporal regulatory processes to sequentially express secretory proteins, of which the mechanism is still largely unknown. Adamson and colleagues [101] showed that *SDS3*, a component of the Sin3 histone deacetylase corepressor complex involved in histone modification and repression of transcription, was particularly downregulated concurrently with a number of expression differences in secretory protein transcripts in *A. maculatum* ticks. The authors proposed that tick secretory proteins might, to some degree, be under epigenetic regulation by histone modification and chromatin remodeling. Based on these results, 34 genes associated with histone modification were identified in the transcriptome of *I. ricinus* [23]. Additionally, recent surveillance of 5 histone and 34 histone modifying enzyme transcripts in *I. scapularis* indicated that *Anaplasma phagocytophilum* altered tick epigenetics to assist pathogen infection and multiplication during infection [102], which changed the expression of the tick’s salivary transcripts [103]. The relationship between epigenetic gene regulation and long non-coding RNA (lncRNA) has been well established in recent years (as reviewed in [104–106]), by which lncRNAs bind to and act as scaffolds between specific sequences in the genome to be transcriptionally regulated and chromatin-modifying enzymes. Long ncRNAs are RNA molecules larger than 200 bp that contain no open reading frames (> 300 bp) for protein translation [107]. These lncRNAs have been largely unexplored in ticks, although a set of 4439 predicted non-coding RNA genes were annotated in the recently completed *I. scapularis* genome [26]. Also, in *R. appendiculatus*, 7414 assembled transcripts contained no predicted ORFs and this set of transcripts represented a striking 40% of the differentially expressed transcripts in the salivary glands [37]. Similarly, in the current transcriptome assembly of *R. zambeziensis,* no open reading frames could be predicted for a third of the assembled transcripts (7947 transcripts). These transcripts represented 16% of the total expression in the salivary glands and most (98%) showed low protein-coding potential [64–66]. These no ORF-containing transcripts also represented 17% (510 transcripts) of the differentially expressed transcripts in *R. zambeziensis* during feeding. This set of transcripts with no predicted ORFs likely contains putative lncRNAs, but also probably protein-coding transcripts for which the predicted ORFs were too small to be retained or potentially misassembled sequences of no biological significance. These protein-coding transcripts are unfortunately not easily distinguishable from lncRNAs and warrants further examination. The large number of differentially expressed transcripts in feeding *R. zambeziensis* ticks, for which no ORFs could be predicted, suggests that lncRNA molecules might be involved in tick blood-feeding, potentially through the transcriptional regulation of tick secretory proteins. This could either be as a complementary mechanism to the proposed epigenetic regulation by Adamson et al. [101] or by other more direct regulatory functions of lncRNAs, such as: acting as transcriptional co-regulators or co-repressors, binding to transcription factors to act as decoys, controlling alternative splice variants, stabilising mRNA by sequestering micro RNAs (miRNAs) away from targets, to name but a few (reviewed in [104, 106]).

The role of lncRNAs in the regulation of the vertebrate immune system has been well established in the last years (reviewed in [108, 109]), although the function in arthropod immunity remains to be determined. Recently, a potential role for lncRNAs in vector immunity has been identified by the increase of a number of *Aedes aegypti* lncRNAs in response to virus and endosymbiont infection [110]. It has also been shown that the vertebrate immune system can be modulated by viral lncRNAs to enhance virus survival during infection of the host [111, 112]. Apart from normal secretory processes, tick salivary glands also excrete molecules into the saliva by a mechanism known as apocrine secretion, where whole pieces of the cells are shed and cytoplasmic contents excreted into the lumen of the acinus [20, 113, 114]. It is conceivable that should it be proven that ticks express lncRNAs that target host defences, this excretion mechanism might be the entry point of the lncRNAs into tick saliva. The functions of lncRNAs in arthropods are still elusive, but the significant number of differentially expressed no ORF-containing transcripts observed in *R. zambeziensis*, the extensive transcriptional regulatory functions described for lncRNAs and the potential host immunomodulation by parasite-derived lncRNAs, warrant further investigations of these important RNA molecules in ticks.

Similar to observations in other *Rhipicephalus* species [34, 37], we found that the Glycine rich family in the transcriptome of *R. zambeziensis* was an exceptionally large contributor (71%) to the total expression of the secretory protein class. Maruyama and colleagues [115] previously postulated that tick species with short mouthparts, e.g. *Rhipicephalus*, require large quantities of Glycine rich proteins to form the cement-cone for adhesion to the host’s skin [3, 116]. The conclusions of the authors [115] were based on only a few species and limited Expression Sequence Tag (EST) data, but recent advances in next generation sequencing of tick transcriptomes have extended the hypothesis to more species with better confidence. Accordingly, the expression contribution of the Glycine rich family to the secretory protein class in ticks with short mouthparts was between 48 and 71% based on three *Rhipicephalus* species ([34, 37], present study) and between 3 and 28% for ticks with long mouthparts based on four *Amblyomma* [31, 33] and a single *Hyalomma* [39] species. *Rhipicephalus* (0.34 mm) has much shorter mouthparts than *Hyalomma* (0.62 mm) and *Amblyomma* (1.27 mm) ticks, with no overlap between the three groups [117]. Another observation in the *R. zambeziensis* transcriptome was that the Glycine rich family was expressed nearly twice as much in male than female ticks (382,258 TPM in males and 202,292 TPM in females), an observation shared with other *Rhipicephalus* species [34, 37]. A large contribution of Glycine rich proteins was observed in early feeding females, followed by downregulation in late feeding, as would be expected for the establishment of the cement-cone, which is generally created within the first three to 4 days after attachment [118]. Contrary, in male salivary glands, Glycine rich proteins remained disproportionally overrepresented in all time points. In light of the cement-cone, the abundance of the Glycine rich family in males is counterintuitive as one would expect female ticks, considering their extended feeding times and exceptional expansion in body size [3], to require a larger cement-cone, and therefore more Glycine rich expression. However, the Glycine rich family is a superfamily to which a number of alternative functions have been ascribed that might be involved during feeding and employed by male ticks to maintain the feeding site. For instance, some Glycine rich proteins have shown to function as antimicrobial peptides in insects (reviewed in [119]) and might assist prolonged tick feeding by keeping the feeding cavity free from microbial infections. Additionally, Glycine rich proteins may also contain RNA-recognition motifs to bind RNA during transcriptional regulation and splicing (reviewed in [120]) or to bind nucleic extrusions from neutrophil extracellular traps of the host defence response (as proposed by Maruyama et al. [115]). Furthermore, in ticks, some Glycine rich proteins have shown to be immune-dominant [79, 121] and might be used by the males as decoy antigens to preoccupy the host defences, thereby assist female feeding [97].

In addition, the *R. zambeziensis* data revealed that even though the expression level of the Glycine rich family was double in males than females, the number of differentially expressed transcripts were considerably fewer: six male and 18 female differentially expressed transcripts for the gender-specific comparison; and 22 male and 89 female differentially expressed transcripts for the feeding-specific comparisons. Similarly, in *R. pulchellus*, two male and 19 female-specific upregulated transcripts were found [34]. These data suggest that females require a more diverse set of Glycine rich proteins in their salivary glands, potentially indicative of a more complex, longer lasting cement-cone that might facilitate the prolonged feeding times and extreme body size expansions seen in female ticks [3]. Alternatively, the Glycine rich protein diversity observed in female ticks might be a mechanism of antigenic variation. Antigenic variation has been proposed as the reason *R. microplus*, a one-host tick that feeds for an extensive amount of time on the same host, showed a larger variety of Glycine rich proteins when compared to two- or three-host ticks [115]. As a final consideration, the nucleotide sequences of the Glycine rich proteins are low in complexity and contain multiple repeats. A technical limitation of assembly algorithms when using short next generation sequencing data, such as Illumina sequences, is the difficulty to assemble low complexity and repeat sequences [122]. This becomes apparent when considering that from all the predicted *R. zambeziensis* proteins only 5% had no predicted methionine start codons, but when assessing only the predicted Glycine rich proteins, 35% had no predicted start codons, indicating a number of fragmented or incomplete sequences in this family. An example of this was seen by the inability to assembly the putative orthologue of RIM36, a Glycine rich cement protein of *R. appendiculatus* [79], into a single transcript. Third generation sequencing technologies, Pacific BioSciences Iso-Seq and Oxford Nanopore MinION sequencing, generate sequence reads with much longer lengths (> 10 Kb) than Illumina sequencing and are used to improve general transcriptome assembly complications, e.g. repeat regions and alternative splice variants [123, 124]. In future, the use of these technologies will assist with the assembly of full-length transcripts for the Glycine rich protein family and tick multi-gene families in general.

Another major contributing family to the transcriptome expression of specifically unfed ticks was the Histamine release factor (HRF) family, representing 38% of female and 13% of male transcript expression in unfed ticks. This family consisted of a single protein, Rzam_Mc198, which showed 73.4% protein identity to the *I. scapularis* tick histamine release factor (tHRF, AAY66972.1) [125]. Tick HRF binds to basophil cells and induces the release of histamine [125, 126], a molecule integral to the vertebrate inflammatory response. Ticks are highly sensitive to histamine during the early stages of feeding, but the sensitivity diminishes towards the end of feeding [127] and histamine becomes required for rapid engorgement presumably by facilitating vascular permeability and blood flow [125]. Contrary to the increasing expression of *tHRF* in *I. scapularis* as feeding progressed [125], a considerable overexpression of *tHRF* was observed in unfed *R. zambeziensis* ticks. Knowing the detrimental effect of histamine on feeding site establishment [127], it is likely that tHRF has a different function prior to feeding, in the free-living stages of *R. zambeziensis*. Histamine release factor proteins are highly conserved and found in a number of eukaryotes and, apart from involvement in inflammation by histamine release, have functions in early developmental processes and cell survival by anti-apoptotic activity in response to stressors such as heat shock and oxidative stress (reviewed in [128]). Desiccation, due to low environmental humidity and high temperatures, is one of the main constraints of free-living ticks surviving the many months between feeding stages, resulting in ticks requiring adaptations to survive in certain climatic regions [129]. *Rhipicephalus zambeziensis* naturally occurs in hot, dry regions with low humidity in southern Africa [40, 50] and has shown high tolerance for extreme experimental abiotic conditions, including low humidity and high temperature [44]. Additionally, the marked wet and dry climatic seasons in southern Africa results in an extended period of inactivity of *R. zambeziensis* where only few of the life stages are found on hosts in the long, dry season [50]. Yet, the tick does not undergo behavioural diapause [130] and it is unclear how it survives these long periods. It is conceivable that the overexpression of *tHRF* in unfed *R. zambeziensis* might contribute to the survival of the tick during free-living and questing periods, potentially by means of increased tolerance to stressors such as water/moisture deficiencies, an association that remains to be experimentally verified.

Another function of tHRF was seen during pathogen transmission, where the *I. scapularis tHRF* gene was upregulated during *Borrelia burgdorferi*-infection. Silencing of the gene by RNA interference or immunisation with recombinant tHRF resulted in reduced transmission rates to the vertebrate host [125], indicating that tHRF functions as a saliva-assisted transmission (SAT) molecule [131]. *Rhipicephalus zambeziensis* and *R. appendiculatus* are vectors of the protozoan parasite, *T. parva* [41, 42], and it has experimentally been shown that *R. zambeziensis* has better vector competence for *T. parva* [45–47]. Integral to the life-cycle of *T. parva* is the tick’s salivary glands where the pathogen multiplies and awaits transmission to the vertebrate host [132] and important vector-pathogen interactions can be expected to occur in these organs. Indeed, the overall level of *tHRF* expression in the salivary glands of *R. zambeziensis*, at 6057 TPM, was about 5-times more than the expression seen for the *R. appendiculatus tHRF* gene, 1212 TPM, generated in a previous study [37]. As tHRF has been shown to function as a SAT molecule enhancing pathogen transmission, the variation in expression between the *tHRFs* of *R. zambeziensis* and *R. appendiculatus* might, at least to some degree, explain the variation observed in the vector competence of *T. parva* of the two tick species.

## Conclusions

In this work we reported the first *de novo* transcriptome assembly of *R. zambeziensis*. The deposition of 13,584 annotated predicted proteins into GenBank vastly improves the sequence availability of the tick and will assist future studies in this largely neglected tick species. As would be expected, an abundance of secretory protein transcription was seen in the salivary glands, the organs in close proximity to the host that are actively expressing proteins to orchestrate host immune modulation. A large number of these secretory transcripts showed significant differential expression resulting in intricate expression profiles that changed considerably over feeding stages. Dynamic secretory protein transcription is possibly a result of antigenic variation, a mechanism previously proposed by which exposed tick antigens are ‘switched’ while evading host immune detection. Interchangeable expression of secretory proteins will have serious implications for recombinant vaccine development, as multi-genic secretory proteins have shown to be functionally redundant. Tick sialotranscriptomes, such as *R. zambeziensis*, will assist recombinant vaccine development by having the sequences of all members of a multi-gene family available to design vaccines against potentially shared antigens. Furthermore, a large number of transcripts for which no ORFs could be predicted were differentially expressed in the *R. zambeziensis* transcriptome, suggesting a role for lncRNAs in tick blood-feeding and specifically, transcriptional regulation of secretory proteins, and motivates further investigation of this potentially important RNA species in ticks. Additionally, the transcriptome will enhance our understanding of tick biology, for example, in this study we observed: data suggesting gender differences in cement-cone complexity and feeding behaviour; a potential candidate gene for survival of free-living *R. zambeziensis* ticks; and a potential molecular explanation for the differences observed in vector competence between two tick species. Understanding the biology of *R. zambeziensis* is of major importance for tick-borne disease control in southern Africa, as the tick is an efficient vector of *T. parva*, well adapted for extreme environments and, with the intensification of climate change, predicted to expand its natural distribution in future.

## Additional files

Additional file 1: Table S1. Specifications of *R. zambeziensis* library preparation procedures and the size and number of sequence reads before and after quality filtering. **Table S2.** Putative *R. zambeziensis* orthologues of previously characterised *R. appendiculatus* proteins. **Table S3.** Expression proportions of the highest contributing secretory protein families during different feeding time points. **Table S4.** Differential expression analysis between female and male *R. zambeziensis* ticks. **Table S5.** Number of differentially expressed transcripts in the protein classes and secretory protein families of *R. zambeziensis* during feeding. (DOCX 80 kb)

Additional file 2: Table S6. Annotation of the *R. zambeziensis* transcriptome. (XLSX 11156 kb)

Additional file 3: Figure S1. Gene Ontology (GO) characterisation of the *R. zambeziensis* transcriptome. Level 2 GO terms of cellular components, molecular functions and biological processes were visualised using WEGO (Web Gene Ontology Annotation Plot). These included 18,436 cellular components, 20,487 biological processes and 9659 molecular functions. **Figure S2.** KOG clustering of *R. zambeziensis* transcripts. In total, 9620 *R. zambeziensis* transcripts were assigned to 25 Eukaryotic Clusters of Orthologs (KOG) functional categories, of which 3814 were unique KOG terms. **Figure S3.** Top 30 most abundant KEGG pathways identified in the *R. zambeziensis* transcriptome. Four thousand eight hundred and sixty nine transcripts were assigned to 338 *I. scapularis* Kyoto Encyclopedia of Genes and Genomes (KEGG) pathways. **Figure S4.** Top 30 Pfam domain occurrences in the *R. zambeziensis* predicted proteins. A total of 13,451 Pfam domains were observed in the *R. zambeziensis* proteins, of which 3601 were unique. Eight thousand and sixty one of the proteins contained at least one Pfam domain. (DOCX 553 kb)

Additional file 4: Table S7. Annotation of the predicted proteins of *R. zambeziensis*. (XLSX 7708 kb)
