# Supplementary material for: Sialotranscriptomics of Rhipicephalus zambeziensis reveals intricate expression profiles of secretory proteins and suggests tight temporal transcriptional regulation during blood-feeding
Source: Parasit Vectors. 2017 Aug 10;10:384. doi: 10.1186/s13071-017-2312-4 (PMC5553602; doi:10.1186/s13071-017-2312-4)
Supplement: Supplementary file 1 — Specifications of R. zambeziensis library preparation procedures and the size and number of sequence reads before and after quality filtering. Table S2. Putative R. zambeziensis orthologues of previously characterised R. appendiculatus proteins. Table S3. Expression proportions of the highest contributing secretory protein families during different feeding time points. Table S4. Differential expression analysis between female and male R. zambeziensis ticks. Table S5. Number of differentially expressed transcripts in the protein classes and secretory protein families of R. zambeziensis during feeding. (DOCX 80 kb) [file 13071_2017_2312_MOESM1_ESM.docx]

**Additional file 1: Tables S1 – S5.**

**Additional file 1: Table S1. Specifications of *R. zambeziensis* library preparation procedures and the size and number of sequence reads before and after quality filtering.**

| Dataset | Library preparation (concentration of starting total RNA) | Library preparation (RNA fragmentation time) | Library preparation (number of amplification cycles) | Library preparation (size selection by excision from agarose gel) | Illumina instrument used for sequencing | Number of raw sequence reads (read 1/ read 2) | Size of raw sequence reads (bp) | Number of quality filtered paired end sequence reads (read 1/ read 2) | Number of quality filtered single end sequence reads | Size range of quality filtered sequence reads (bp) | Percentage of reads retained (paired end formation/ single end formation) |
| --- | --- | --- | --- | --- | --- | --- | --- | --- | --- | --- | --- |
| **HiScanSQ generated sequence reads** | | |  |  |  |  |  |  |  |  |  |
| F0: Female - unfed | 2 ug | 8 min | 12 | ±300 bp | HiScanSQ | 32027583/ 32027583 | 100 | 25391810/ 25391810 | 5007301 | 50-100 | 79.3/ 7.8 |
| F3: Female - 3 days feeding | 2 ug | 8 min | 12 | ±300 bp | HiScanSQ | 32142442/ 32142442 | 100 | 25321431/ 25321431 | 5123605 | 50-100 | 78.8/ 8.0 |
| F5: Female - 5 days feeding | 2 ug | 8 min | 12 | ±300 bp | HiScanSQ | 37528275/ 37528275 | 100 | 29918808/ 29918808 | 5786823 | 50-100 | 79.7/ 7.7 |
| M0: Male - unfed | 2 ug | 8 min | 12 | ±300 bp | HiScanSQ | 27591382/ 27591382 | 100 | 23335530/ 23335530 | 3236827 | 50-100 | 84.6/ 5.9 |
| M3: Male - 3 days feeding | 2 ug | 8 min | 12 | ±300 bp | HiScanSQ | 26047465/ 26047465 | 100 | 21694635/ 21694635 | 3232318 | 50-100 | 83.3/ 6.2 |
| M5: Male - 5 days feeding | 2 ug | 8 min | 12 | ±300 bp | HiScanSQ | 22145769/ 22145769 | 100 | 18518042/ 18518042 | 2702203 | 50-100 | 83.6/ 6.1 |
| **Total HiScanSQ sequence data** |  |  |  |  |  | **177482916/ 177482916** | **100** | **144180256/ 144180256** | **25089077** | **50-100** | **81.2/ 7.1** |
| **MiSeq generated sequence reads** | | |  |  |  |  |  |  |  |  |  |
| Equimolar mix of all six samples | 2 ug | 3 min^a^ | 12 | ±600 - 1000 bp | MiSeq | 22653340/ 22653340 | 300 | 6901796/ 6901796 | 15631696 | 50-300 | 30.5/ 34.5 |
| Merging of paired end MiSeq reads^b^ |  |  |  |  |  | 6901796/ 6901796 | 50-300 | 2254236/ 2254236 | 4647560 | 50-580 | 32.7/ 67.3 |
| **Total MiSeq sequence data** |  |  |  |  |  | **22653340/ 22653340** | **300** | **2254236/ 2254236** | **20279256** | **50-580** | **10.0/ 44.8** |
| **Total generated sequence reads** | | |  |  |  |  |  |  |  |  |  |
| **Total sequence data** | | |  |  |  | **200136256/ 200136256** | **100-300** | **146434492/ 146434492** | **45368333** | **50-580** | **73.2/ 11.3** |

^a^ Varying RNA fragmentation time was used during the preparation of the MiSeq sequencing library to facilitate the generation of larger fragments for sequencing on the longer read Miseq instrument.

^b^ Due to the long sequence reads generated by the Miseq instrument many of the paired end sequences overlapped and were merged into a single read when an overlap of 20 bp was observed.

**Additional file 1: Table S2. Putative *R. zambeziensis* orthologues of previously characterised *R. appendiculatus* proteins.**

| **Protein name** | **Protein description** | **Accession number** | **Reference^a^** | ***R. zambeziensis* Protein ID** | **Identity (%)** | **Full-length^b^** | **Combined TPM^d^** | **Female TPM^d^** | **Male TPM^d^** |
| --- | --- | --- | --- | --- | --- | --- | --- | --- | --- |
| IGBP-MA | Immunoglobulin G binding protein-Male A | AAB68801.1 | [77] | Rzam_Mc5608 | 97 | Complete | 2113.1 | 1.1 | 4076.8 |
| IGBP-MB | Immunoglobulin G binding protein-Male B | AAB68802.1 | [77] | Rzam_Mc3335 | 92 | Complete | 2588.6 | 1.1 | 5065.3 |
| IGBP-MC | Immunoglobulin G binding protein-Male C | AAB68803.1 | [77] | Rzam_Mc3213 | 98 | Complete | 4434.2 | 1.5 | 8720.0 |
| HBP1 | Female-specific histamine-binding protein 1 | O77420 | [78] | Rzam_Mc9434 | 91 | Complete | 250.3 | 512.5 | 0 |
| HBP2 | Female-specific histamine-binding protein 2 | O77421 | [78] | Rzam_Mc5492 | 75 | Complete | 11.6 | 24.0 | 0 |
| HBPM | Male-specific histamine-binding salivary protein | O77422 | [78] | Rzam_Mc12946 | 42 | Complete | 24.4 | 0 | 47.4 |
| RIM36 | *Rhipicephalus* immuno-dominant molecule 36 | AAK98794.1 | [79] | Rzam_Mc6473 | 98 | Fragment^c^ | 10590.3 | 5205.0 | 15850.2 |
|  |  |  |  | Rzam_Mc186 | 96 | Fragment^c^ | 6645.5 | 2908.9 | 10290.5 |
| 64P | Salivary gland-associated protein 64P | AAM09648.1 | [80] | Rzam_Mc354 | 85 | Complete | 497.5 | 95.3 | 884.3 |
| RAS-1 | *R. appendiculatus* serine proteinase inhibitor serpin-1 | AAK61375.1 | [81] | Rzam_Mc7211 | 90 | Complete | 15.6 | 7.0 | 23.7 |
| RAS-2 | *R. appendiculatus* serine proteinase inhibitor serpin-2 | AAK61376.1 | [81] | Rzam_Mc9158 | 90 | Fragment | 7.8 | 9.7 | 6.1 |
| RAS-3 | *R. appendiculatus* serine proteinase inhibitor serpin-3 | AAK61377.1 | [81] | Rzam_Mc8493 | 94 | Complete | 39.7 | 69.9 | 10.3 |
| RAS-4 | *R. appendiculatus* serine proteinase inhibitor serpin-4 | AAK61378.1 | [81] | Rzam_Mc5334 | 71 | Complete | 14.8 | 29.4 | 0.6 |
| TdP1 | Tryptase inhibitor precursor | AAW32666.1 | [82] | Rzam_Mc366 | 34 | Complete | 49.5 | 92.1 | 9.4 |
| Ra-KLP | *R. appendiculatus* Kunitz/BPTI-like protein | ACM86785.1 | [83] | Rzam_Mc262 | 86 | Complete | 4.4 | 9.1 | 0 |
| JL-RA1 | Japanin-like-RA1 precursor | AGF70151.1 | [84] | Rzam_Mc2302 | 90 | Complete | 1.8 | 3.6 | 0.1 |
| JL-RA2 | Japanin-like-RA2 precursor | AGF70152.1 | [84] | Rzam_Mc3898 | 58 | Complete | 1.7 | 2.1 | 1.4 |
| Japanin | Japanin precursor | AGF70149.1 | [84] | Rzam_Mc597 | 32 | Complete | 3.2 | 6.6 | 0 |

^a^ Referencing based on numbering in manuscript.

^b^ Full-length based on the presence of a predicted start and stop codon in the deduced amino acid sequence.

^c^ RIM36 was assembled into two separate transcripts, each coding for its own fragmented open reading frame.

^d^ Expression was estimated as TPM (transcripts per million).

**Additional file 1: Table S3. Expression proportions of the highest contributing secretory protein families during different feeding time points.**

|  | Female day 0 (TPM) | Female day 0 (%) | Female day 3 (TPM) | Female day 3 (%) | Female day 5 (TPM) | Female day 5 (%) | Male day 0 (TPM) | Male day 0 (%) | Male day 3 (TPM) | Male day 3 (%) | Male day 5 (TPM) | Male day 5 (%) |
| --- | --- | --- | --- | --- | --- | --- | --- | --- | --- | --- | --- | --- |
| Glycine rich superfamily | 22529,8 | 35,66 | 425477,1 | 75,20 | 47415,7 | 18,00 | 86953,4 | 67,12 | 526364,9 | 84,26 | 411893,1 | 71,77 |
| Histamine release factor | 23926,9 | 37,88 | 705,4 | 0,12 | 2830,4 | 1,07 | 17073,3 | 13,18 | 538,3 | 0,09 | 792,2 | 0,14 |
| Lipocalin | 2758,9 | 4,37 | 13092,3 | 2,31 | 68973,3 | 26,18 | 5059,6 | 3,91 | 31440,1 | 5,03 | 15824,5 | 2,76 |
| Transport and catabolism | 1768,9 | 2,80 | 327,6 | 0,06 | 1052,2 | 0,40 | 1684,1 | 1,30 | 318,1 | 0,05 | 654,1 | 0,11 |
| Mucin | 1499,0 | 2,37 | 2301,2 | 0,41 | 8328,5 | 3,16 | 1600,8 | 1,24 | 1421,9 | 0,23 | 3041,1 | 0,53 |
| Bovine pancreatic trypsin inhibitor | 1155,3 | 1,83 | 7072,8 | 1,25 | 27400,5 | 10,40 | 2808,4 | 2,17 | 12669,7 | 2,03 | 10024,5 | 1,75 |
| Reprolysin | 1093,6 | 1,73 | 5051,5 | 0,89 | 9538,2 | 3,62 | 1256,0 | 0,97 | 2436,6 | 0,39 | 4853,2 | 0,85 |
| Folding, sorting and degradation (including Cathepsins) | 1076,0 | 1,70 | 1367,4 | 0,24 | 4347,2 | 1,65 | 1438,8 | 1,11 | 2151,0 | 0,34 | 13834,8 | 2,41 |
| Basic tail secreted protein | 984,9 | 1,56 | 6464,1 | 1,14 | 13846,5 | 5,26 | 1811,5 | 1,40 | 2425,1 | 0,39 | 2953,6 | 0,51 |
| 24 kDa family | 848,4 | 1,34 | 645,1 | 0,11 | 200,7 | 0,08 | 934,6 | 0,72 | 321,1 | 0,05 | 373,1 | 0,07 |
| TIL domain | 597,1 | 0,95 | 9405,6 | 1,66 | 2612,1 | 0,99 | 1982,0 | 1,53 | 5756,7 | 0,92 | 16662,8 | 2,90 |
| Secretory - unknown function | 524,1 | 0,83 | 66351,4 | 11,73 | 8185,8 | 3,11 | 1683,1 | 1,30 | 13897,2 | 2,22 | 13318,4 | 2,32 |
| Defensin | 7,3 | 0,01 | 11841,0 | 2,09 | 3973,6 | 1,51 | 83,8 | 0,06 | 3099,7 | 0,50 | 1694,7 | 0,30 |
| 8.9 kDa family | 274,6 | 0,43 | 4540,0 | 0,80 | 30232,9 | 11,47 | 470,5 | 0,36 | 3131,9 | 0,50 | 3513,3 | 0,61 |
| Ixodegrin B | 148,1 | 0,23 | 1629,0 | 0,29 | 8695,3 | 3,30 | 209,2 | 0,16 | 253,7 | 0,04 | 282,7 | 0,05 |
| 28 kDa Metastriate family | 137,9 | 0,22 | 662,9 | 0,12 | 7921,2 | 3,01 | 141,9 | 0,11 | 1547,3 | 0,25 | 559,2 | 0,10 |
| ML domain | 126,2 | 0,20 | 38,7 | 0,01 | 143,4 | 0,05 | 118,8 | 0,09 | 4300,9 | 0,69 | 36254,4 | 6,32 |
| Immunoglobulin G binding protein A | 0,0 | 0,00 | 5,2 | 0,00 | 1,2 | 0,00 | 2,5 | 0,00 | 4487,0 | 0,72 | 23233,5 | 4,05 |
| Other secretory proteins | 3716,1 | 5,88 | 8822,4 | 1,56 | 17780,8 | 6,75 | 4236,9 | 3,27 | 8107,6 | 1,30 | 14176,4 | 2,47 |
| Total secretory protein portion | 63173,1 | 100 | 565800,4 | 100 | 263479,3 | 100 | 129549,1 | 100 | 624668,7 | 100 | 573939,3 | 100 |

The expression level (measured in transcripts per million, TPM) and proportion (%) of each secretory protein family was estimated per timepoint. Proportions were visual representated in Fig. 3 of the manuscript.

**Additional file 1: Table S4. Differential expression analysis between female and male *R. zambeziensis* ticks.**

| Protein classes/ families | Female up-regulated | Male up-regulated | χ2 | p-value |
| --- | --- | --- | --- | --- |
| *Secretory protein class* | *376* | *259* | *21.89* | < 0.0001*** |
| Lipocalin | 140 | 42 | 52.77 | < 0.0001* |
| Digestive system (including Serine proteases) | 0 | 41 | 41.00 | < 0.0001* |
| Cystatin | 0 | 26 | 26.00 | < 0.0001* |
| Reprolysin | 27 | 3 | 19.20 | < 0.0001* |
| TIL domain | 4 | 29 | 18.94 | < 0.0001* |
| 28 kDa Metastriate family | 23 | 2 | 17.64 | < 0.0001* |
| Folding, sorting and degradation (including Cathepsins) | 1 | 17 | 14.22 | 0.0002* |
| 7DB family | 0 | 13 | 13.00 | 0.0003* |
| 8.9 kDa family | 36 | 12 | 12.00 | 0.0005* |
| DA-P36 family | 12 | 1 | 9.31 | 0.0023 |
| Bovine pancreatic trypsin inhibitor | 37 | 17 | 7.41 | 0.0065 |
| Ixodegrin B | 10 | 1 | 7.36 | 0.0067 |
| One of each family | 7 | 0 | 7.00 | 0.0082 |
| Glycine rich superfamily | 18 | 6 | 6.00 | 0.0143 |
| Gluzincin | 8 | 1 | 5.44 | 0.0196 |
| Immunoglobulin G binding protein A | 0 | 5 | 5.00 | 0.0253 |
| ML domain | 0 | 4 | 4.00 | 0.0455 |
| Mucin | 0 | 4 | 4.00 | 0.0455 |
| Hirudin | 3 | 0 | 4.00 | 0.0455 |
| Chitin-binding proteins | 0 | 3 | 3.00 | 0.0833 |
| Carboxypeptidase inhibitor | 3 | 0 | 3.00 | 0.0833 |
| Evasin | 12 | 5 | 2.88 | 0.0896 |
| Antigen 5 family | 0 | 2 | 2.00 | 0.1573 |
| Basic tail secreted protein | 13 | 8 | 1.19 | 0.2752 |
| 5'-Nucleotidase | 1 | 3 | 1.00 | 0.3173 |
| 8 kDa Amblyomma family | 0 | 1 | 1.00 | 0.3173 |
| Sphingomyelinase | 0 | 1 | 1.00 | 0.3173 |
| 24 kDa family | 1 | 0 | 1.00 | 0.3173 |
| Dermacentor 9 kDa expansion | 1 | 0 | 1.00 | 0.3173 |
| Glycan biosynthesis and metabolism | 1 | 0 | 1.00 | 0.3173 |
| Kazal domain | 1 | 0 | 1.00 | 0.3173 |
| Lipid metabolism | 1 | 0 | 1.00 | 0.3173 |
| Serine/threonine protein kinase | 1 | 0 | 1.00 | 0.3173 |
| Secretory - unknown function | 9 | 6 | 0.60 | 0.4386 |
| Transport and catabolism | 1 | 2 | 0.33 | 0.5637 |
| Defensin | 2 | 1 | 0.33 | 0.5637 |
| Microplusin | 2 | 2 | 0.00 | 1.0 |
| Serpin | 1 | 1 | 0.00 | 1.0 |
| *Housekeeping protein class* | *87* | *69* | *2.08* | *0.1495* |
| *Unknown function protein class* | *56* | *91* | *8.33* | *0.0039* |
| *No hit protein class* | *21* | *26* | *0.53* | *0.4658* |
| *Transcripts without predicted ORFs* | *101* | *123* | *2.16* | *0.1416* |
| **Total** | **642** | **568** | **4.53** | **0.0334** |

Differential expression analysis was performed using the edgeR (Empirical analysis of digital gene expression data in R) software package (parameters: fixed dispersion of 0.4, fold change of >4 and FDR p-value of < 0.01).

Chi-square test was performed to compare differences between female and male ticks. χ2 - values and p-values are indicated. df = 1.

* Significant Chi-square test (Bonferroni corrected p-value < 0.00132).

**Additional file 1: Table S5. Number of differentially expressed transcripts in the protein classes and secretory protein families of *R. zambeziensis* during feeding.**

| Protein classes/ families | F0vsF3 (F0 Up) | F0vsF3 (F3 Up) | F0vsF5 (F0 Up) | F0vsF5 (F5 Up) | F3vsF5 (F3 Up) | F3vsF5 (F5 Up) | M0vsM3 (M0 Up) | M0vsM3 (M3 Up) | M0vsM5 (M0 Up) | M0vsM5 (M5 Up) | M3vsM5 (M3 Up) | M3vsM5 (M5 Up) |
| --- | --- | --- | --- | --- | --- | --- | --- | --- | --- | --- | --- | --- |
| *Secretory protein class* | *15* | *541* | *111* | *522* | *411* | *103* | *4* | *335* | *4* | *367* | *0* | *2* |
| Lipocalin | 1 | 134 | 21 | 150 | 108 | 47 | 0 | 72 | 0 | 50 | 0 | 1 |
| Glycine rich superfamily | 0 | 61 | 1 | 17 | 60 | 1 | 0 | 21 | 0 | 17 | 0 | 0 |
| Reprolysin | 1 | 55 | 18 | 70 | 42 | 4 | 1 | 20 | 0 | 26 | 0 | 0 |
| Bovine pancreatic trypsin inhibitor | 3 | 51 | 31 | 64 | 53 | 7 | 0 | 36 | 0 | 24 | 0 | 0 |
| TIL domain | 0 | 38 | 4 | 11 | 36 | 2 | 0 | 34 | 0 | 37 | 0 | 0 |
| 8.9 kDa family | 0 | 33 | 1 | 34 | 13 | 8 | 0 | 15 | 0 | 16 | 0 | 0 |
| Basic tail secreted protein | 0 | 30 | 4 | 29 | 12 | 4 | 0 | 14 | 0 | 16 | 0 | 0 |
| 28 kDa Metastriate family | 1 | 22 | 3 | 30 | 6 | 5 | 0 | 3 | 0 | 5 | 0 | 0 |
| Gluzincin | 0 | 5 | 5 | 16 | 4 | 8 | 0 | 1 | 0 | 2 | 0 | 0 |
| Evasin | 0 | 17 | 0 | 17 | 5 | 2 | 0 | 6 | 0 | 7 | 0 | 0 |
| Secretory - unknown function | 1 | 12 | 3 | 8 | 10 | 1 | 0 | 8 | 0 | 10 | 0 | 0 |
| Ixodegrin B | 0 | 10 | 2 | 2 | 8 | 0 | 0 | 2 | 0 | 2 | 0 | 0 |
| Carboxypeptidase inhibitor | 0 | 10 | 0 | 2 | 12 | 0 | 0 | 4 | 0 | 3 | 0 | 0 |
| Defensin | 0 | 9 | 0 | 7 | 3 | 0 | 0 | 9 | 0 | 6 | 0 | 0 |
| Mucin | 0 | 4 | 1 | 1 | 11 | 0 | 1 | 4 | 0 | 4 | 0 | 0 |
| DA-P36 family | 1 | 3 | 2 | 12 | 1 | 7 | 0 | 1 | 0 | 1 | 0 | 0 |
| One of each family | 0 | 1 | 0 | 9 | 0 | 3 | 0 | 0 | 0 | 0 | 0 | 0 |
| Serpin | 0 | 1 | 0 | 7 | 0 | 0 | 0 | 1 | 0 | 5 | 0 | 0 |
| Hirudin | 0 | 3 | 0 | 3 | 0 | 0 | 0 | 0 | 0 | 0 | 0 | 0 |
| Thyropin | 0 | 3 | 0 | 4 | 0 | 0 | 0 | 0 | 0 | 0 | 0 | 0 |
| Glycan biosynthesis and metabolism | 1 | 3 | 1 | 3 | 0 | 0 | 0 | 0 | 1 | 0 | 0 | 0 |
| Dermacentor 9 kDa expansion | 0 | 1 | 0 | 2 | 2 | 1 | 0 | 2 | 0 | 2 | 0 | 0 |
| Fibrinogen-related domain | 0 | 6 | 0 | 2 | 1 | 0 | 0 | 4 | 0 | 4 | 0 | 0 |
| Folding, sorting and degradation (including Cathepsins) | 1 | 0 | 0 | 2 | 3 | 0 | 1 | 16 | 1 | 17 | 0 | 0 |
| Kazal domain | 0 | 0 | 0 | 2 | 0 | 1 | 0 | 0 | 0 | 0 | 0 | 0 |
| Lipid metabolism | 0 | 1 | 0 | 2 | 1 | 0 | 0 | 0 | 0 | 0 | 0 | 0 |
| Microplusin | 0 | 3 | 1 | 2 | 0 | 0 | 0 | 2 | 0 | 1 | 0 | 0 |
| SALP15/Ixostatin | 0 | 0 | 0 | 2 | 0 | 0 | 0 | 1 | 0 | 1 | 0 | 0 |
| 24 kDa family | 0 | 3 | 5 | 1 | 1 | 0 | 0 | 0 | 0 | 2 | 0 | 0 |
| 5'-Nucleotidase | 0 | 0 | 1 | 1 | 3 | 0 | 0 | 0 | 0 | 0 | 0 | 0 |
| Antigen 5 family | 0 | 2 | 0 | 1 | 1 | 0 | 0 | 4 | 0 | 4 | 0 | 0 |
| Chitin-binding proteins | 0 | 0 | 0 | 1 | 0 | 0 | 0 | 6 | 0 | 6 | 0 | 0 |
| Cystatin | 0 | 4 | 0 | 1 | 4 | 0 | 0 | 14 | 0 | 26 | 0 | 0 |
| Kazal/SPARC domain | 0 | 1 | 0 | 1 | 0 | 0 | 0 | 0 | 0 | 0 | 0 | 0 |
| ML domain | 0 | 0 | 0 | 1 | 0 | 0 | 0 | 3 | 0 | 4 | 0 | 0 |
| Phospholipase A2 | 0 | 3 | 0 | 1 | 2 | 0 | 0 | 1 | 0 | 1 | 0 | 0 |
| Signal transduction | 0 | 0 | 0 | 1 | 0 | 0 | 0 | 0 | 0 | 0 | 0 | 0 |
| Serine/threonine protein kinase | 0 | 0 | 0 | 1 | 0 | 1 | 0 | 0 | 0 | 0 | 0 | 0 |
| Transport and catabolism | 0 | 1 | 0 | 1 | 0 | 0 | 0 | 2 | 0 | 2 | 0 | 0 |
| Translation | 1 | 1 | 1 | 1 | 0 | 0 | 0 | 0 | 0 | 0 | 0 | 0 |
| 8 kDa Amblyomma family | 0 | 3 | 0 | 0 | 6 | 0 | 0 | 3 | 0 | 3 | 0 | 0 |
| Digestive system (including Serine proteases) | 0 | 4 | 1 | 0 | 3 | 0 | 0 | 12 | 0 | 41 | 0 | 1 |
| Immunoglobulin G binding protein A | 0 | 1 | 0 | 0 | 0 | 0 | 0 | 5 | 0 | 5 | 0 | 0 |
| 14 kDa family | 0 | 1 | 0 | 0 | 0 | 0 | 0 | 0 | 0 | 0 | 0 | 0 |
| Madanin | 0 | 1 | 0 | 0 | 0 | 0 | 0 | 1 | 0 | 1 | 0 | 0 |
| Metalloprotease | 1 | 0 | 0 | 0 | 0 | 0 | 0 | 0 | 1 | 0 | 0 | 0 |
| Sphingomyelinase | 2 | 0 | 5 | 0 | 0 | 0 | 1 | 1 | 0 | 1 | 0 | 0 |
| Transcription | 1 | 0 | 0 | 0 | 0 | 1 | 0 | 0 | 1 | 0 | 0 | 0 |
| 7DB family | 0 | 0 | 0 | 0 | 0 | 0 | 0 | 7 | 0 | 15 | 0 | 0 |
| *Housekeeping protein class* | *53* | *126* | *79* | *219* | *66* | *36* | *47* | *65* | *23* | *72* | *0* | *1* |
| *Unknown function protein class* | *8* | *95* | *38* | *64* | *126* | *11* | *2* | *95* | *3* | *98* | *0* | *2* |
| *No hit protein class* | *4* | *29* | *13* | *26* | *26* | *5* | *3* | *32* | *1* | *28* | *0* | *0* |
| *Transcripts without predicted ORFs* | *23* | *136* | *55* | *149* | *110* | *29* | *7* | *96* | *5* | *116* | *0* | *0* |
| ***Total*** | **103** | **927** | **296** | **980** | **739** | **184** | **63** | **623** | **36** | **681** | **0** | **5** |

Differential expression analysis was performed using the edgeR (Empirical analysis of digital gene expression data in R) software package (parameters: fixed dispersion of 0.4, fold change of >4 and FDR p-value of < 0.01).
