# Supplementary material for: Sialotranscriptomics of Rhipicephalus zambeziensis reveals intricate expression profiles of secretory proteins and suggests tight temporal transcriptional regulation during blood-feeding
Source: Parasit Vectors. 2017 Aug 10;10:384. doi: 10.1186/s13071-017-2312-4 (PMC5553602; doi:10.1186/s13071-017-2312-4)
Supplement: Supplementary file 3 — Gene Ontology (GO) characterisation of the R. zambeziensis transcriptome. Level 2 GO terms of cellular components, molecular functions and biological processes were visualised using WEGO (Web Gene Ontology Annotation Plot). These included 18,436 cellular components, 20,487 biological processes and 9659 molecular functions. Figure S2. KOG clustering of R. zambeziensis transcripts. In total, 9620 R. zambeziensis transcripts were assigned to 25 Eukaryotic Clusters of Orthologs (KOG) functional categories, of which 3814 were unique KOG terms. Figure S3. Top 30 most abundant KEGG pathways identified in the R. zambeziensis transcriptome. Four thousand eight hundred and sixty nine transcripts were assigned to 338 I. scapularis Kyoto Encyclopedia of Genes and Genomes (KEGG) pathways. Figure S4. Top 30 Pfam domain occurrences in the R. zambeziensis predicted proteins. A total of 13,451 Pfam domains were observed in the R. zambeziensis proteins, of which 3601 were unique. Eight thousand and sixty one of the proteins contained at least one Pfam domain. (DOCX 553 kb) [file 13071_2017_2312_MOESM3_ESM.docx]

**Additional file 3: Figures S1 – S4**


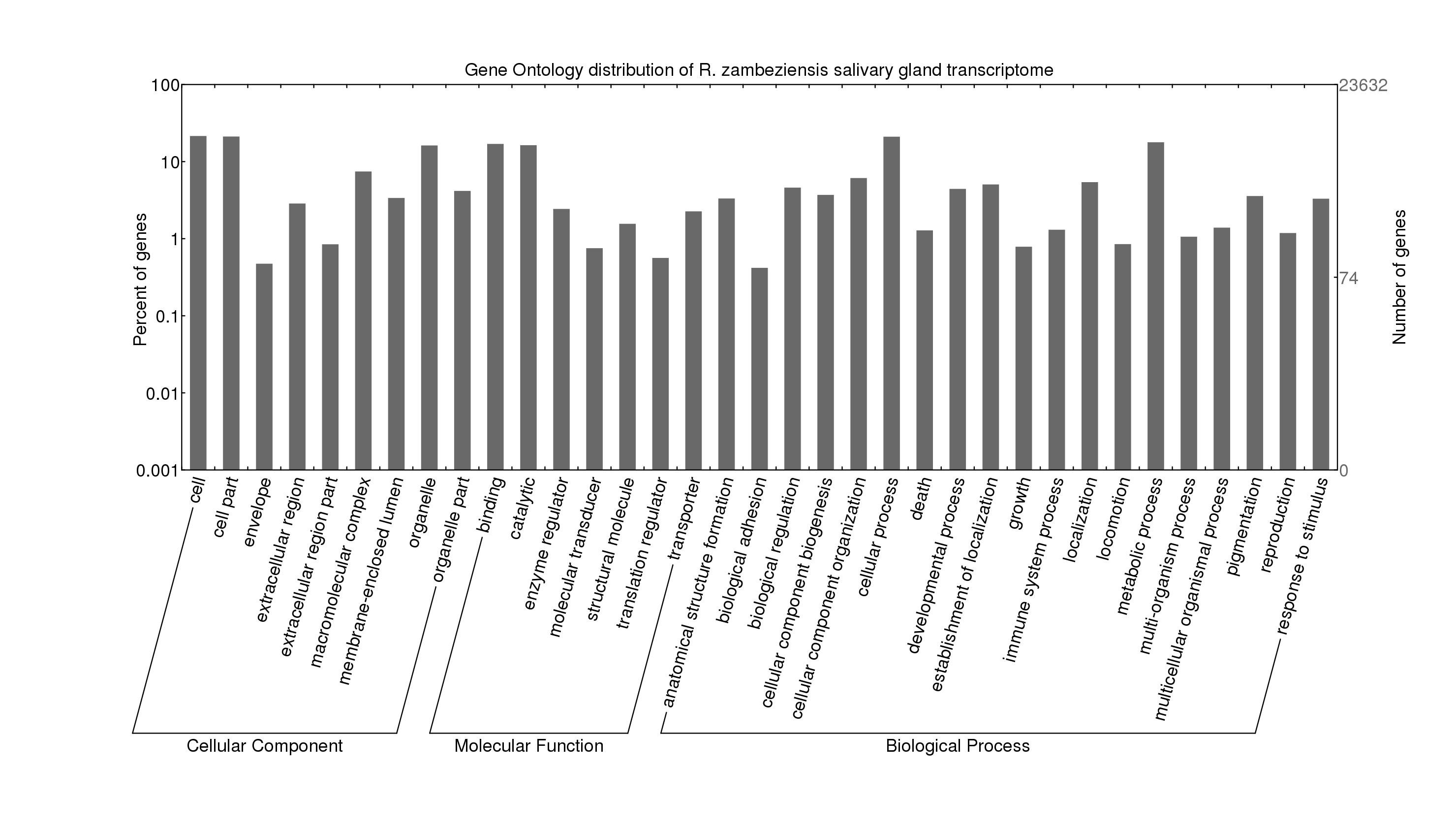


**Additional file 3: Figure S1. Gene Ontology (GO) characterisation of the *R. zambeziensis* transcriptome.** Level 2 GO terms of cellular components, molecular functions and biological processes were visualised using WEGO (Web Gene Ontology Annotation Plot). These included 18 436 cellular components, 20 487 biological processes and 9659 molecular functions.

**Additional file 3: Figure S2. KOG clustering of *R. zambeziensis* transcripts.** In total, 9620 *R. zambeziensis* transcripts were assigned to 25 Eukaryotic Clusters of Orthologs (KOG) functional categories, of which 3814 were unique KOG terms**.**

**Additional file 3: Figure S3.** Top 30 most abundant KEGG pathways identified in the R. zambeziensis transcriptome. Four thousand eight hundred and sixty nine transcripts were assigned to 338 I. scapularis Kyoto Encyclopedia of Genes and Genomes (KEGG) pathways.

**Additional file 3: Figure S4. Top 30 Pfam domain occurrences in the *R. zambeziensis* predicted proteins.** A total of 13 451 Pfam domains were observed in the *R. zambeziensis* proteins, of which 3601 were unique. Eight thousand and sixty one of the proteins contained at least one Pfam domain.
